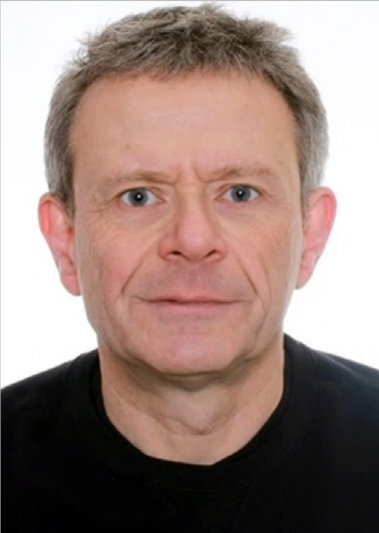# Understanding the Interaction of Polyelectrolyte Architectures with Proteins and Biosystems

**DOI:** 10.1002/anie.202006457

**Published:** 2020-10-27

**Authors:** Katharina Achazi, Rainer Haag, Matthias Ballauff, Jens Dernedde, Jayachandran N. Kizhakkedathu, Dusica Maysinger, Gerd Multhaup

**Affiliations:** ^1^ Institut für Chemie und Biochemie Freie Universität Berlin Takustrasse 3 14195 Berlin Germany; ^2^ IRIS Adlershof Humboldt Universität zu Berlin Zum Grossen Windkanal 6 12489 Berlin Germany; ^3^ Charité-Universitätsmedizin Berlin Institute of Laboratory Medicine Clinical Chemistry, and Pathobiochemistry CVK Augustenburger Platz 1 13353 Berlin Germany; ^4^ Centre for Blood Research Department of Pathology and Laboratory Medicine Life Science Institute Department of Chemistry School of Biomedical Engineering University of British Columbia Vancouver V6T 1Z3 Canada; ^5^ Department of Pharmacology and Therapeutics McGill University Montreal H3G 1Y6 Canada

**Keywords:** complementary binding, counterion release, heparin, inflammation, polyelectrolytes

## Abstract

The counterions neutralizing the charges on polyelectrolytes such as DNA or heparin may dissociate in water and greatly influence the interaction of such polyelectrolytes with biomolecules, particularly proteins. In this Review we give an overview of studies on the interaction of proteins with polyelectrolytes and how this knowledge can be used for medical applications. Counterion release was identified as the main driving force for the binding of proteins to polyelectrolytes: Patches of positive charge become multivalent counterions of the polyelectrolyte and lead to the release of counterions from the polyelectrolyte and a concomitant increase in entropy. This is shown from investigations on the interaction of proteins with natural and synthetic polyelectrolytes. Special emphasis is paid to sulfated dendritic polyglycerols (dPGS). The Review demonstrates that we are moving to a better understanding of charge–charge interactions in systems of biological relevance. Research along these lines will aid and promote the design of synthetic polyelectrolytes for medical applications.

## Graphical abstract

Polyelectrolytes such as DNA or heparin are long linear or branched macromolecules onto which charges are appended. The counterions neutralizing these charges can dissociate in water and this will largely determine the interaction of such polyelectrolytes with biomolecules, particularly with proteins. This Review discusses studies on the interaction of proteins with polyelectrolytes and how this knowledge can be used for medical applications.

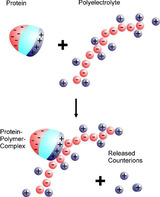

## 1 Introduction

Polyelectrolytes consist of long linear or branched macromolecules that contain charged units. When dispersed in water or a sufficiently polar solvent, the counterions balancing the charge of the polyelectrolyte will partially dissociate. Therefore, the properties of polyelectrolytes in solution will be greatly determined by their counterions. Polyelectrolytes are ubiquitous in biological systems and play a central role in almost all biochemical processes.^[1]^ DNA is perhaps the best‐studied natural polyelectrolyte and in the preceding 50 years most work has been directed towards a detailed understanding of the interaction between DNA and proteins related to DNA repair proteins or transcription factors.[^2^, ^3^, ^4^, ^5^, ^6^, ^7^, ^8^, ^9^, ^10^, ^11^] The thermodynamics of the binding of DNA or RNA to proteins has been shown to be dominated by charge–charge interactions, and the biological activity of natural polyelectrolytes such as DNA is intimately related to their highly charged molecular structures.[^2^, ^12^, ^13^, ^14^, ^15^] Heparin provides another example of a natural polyelectrolyte with four charges per repeating unit that has been studied intensively during the last 30 years.[^16^, ^17^, ^18^, ^19^]

Synthetic polyelectrolytes, on the other hand, have become valuable tools for various medical purposes during the past 20 years. Thus, complexes of synthetic polyelectrolytes with DNA are now used as nonviral vectors for gene delivery.[^20^, ^21^] Research along this line has been aimed at well‐defined complexes with optimized efficiency. More recently, block copolymers containing cationic sequences have been used for this purpose, and transfection using polycations is an active field of polymer science these days.[^22^, ^23^, ^24^, ^25^] Other polymer architectures used so far include nanogels,[^26^, ^27^, ^28^, ^29^] which consist of crosslinked polyelectrolytes.[^30^, ^31^] These systems have also become another highly useful device for gene delivery[^21^, ^25^, ^26^, ^32^, ^33^] as well as for the defined uptake and delivery of proteins and drugs in general.[^23^, ^25^]

An equally fascinating and rather recent development is the use of polyelectrolytes as drugs themselves.^[34]^ Here, sulfated dendritic polyglycerol sulfate (dPGS), which consists of a dendritic poly(glycerol) scaffold with each sulfated end group bearing a negative charge, has become a focus of our research.^[35]^ First designed as a replacement for heparin,^[34]^ dPGS has been used for a variety of biomedical purposes that range from tumor targeting to anti‐inflammatory treatment.[^34^, ^36^] Previous studies suggest that the interaction of dPGS with various proteins and cell‐surface molecules proceeds in a specific way. Thus, Dernedde et al.^[36]^ surmised that dPGS can block the cell adhesion molecules (CAMs) L‐ and P‐selectin on leukocytes and activated endothelial cells, respectively, which are central to inflammatory processes, through a selective charge–charge interaction. Hence, dPGS seems to act as a macromolecular inhibitor that may mimic naturally occurring ligands. Prompted by the success of dPGS as an anti‐inflammatory compound, a number of structures have been synthesized that contain dPGS as a building block for various biological processes in which inflammation plays a central role: Nanogels based on dPGS with different degrees of flexibility have been shown to possess antiviral properties.^[37]^ dPGS has also been used as a building block for micellar structures that can be used for targeting tumor cells.^[38]^ Furthermore, its interaction with neural microglia has been the subject of several studies.[^39^, ^40^] Substituted polyglycerols bearing positive charges have been introduced as potent antibleeding agents with excellent anticoagulant reversal activity upon binding the polyanion heparin.[^41^, ^42^, ^43^]

Summing up all the research done to date, it is fair to state that a large number of charged polymeric systems and potential drugs have been synthesized recently and the possible medical applications of these systems are hard to overlook.[^23^, ^25^] However, only a small subset of polyelectrolyte systems has reached the stage of clinical trials. The problems at hand are: Such polymeric drugs must remain active in the complex environment of cells or a multitude of proteins in the blood stream. Ideally, the drug should interact only with a chosen target structure in a highly specific manner. Unspecific interaction with blood proteins should be avoided. At this moment, we clearly lack a general understanding of these systems, which would allow us to design them in a straightforward manner to circumvent these problems.

**Figure 1:**
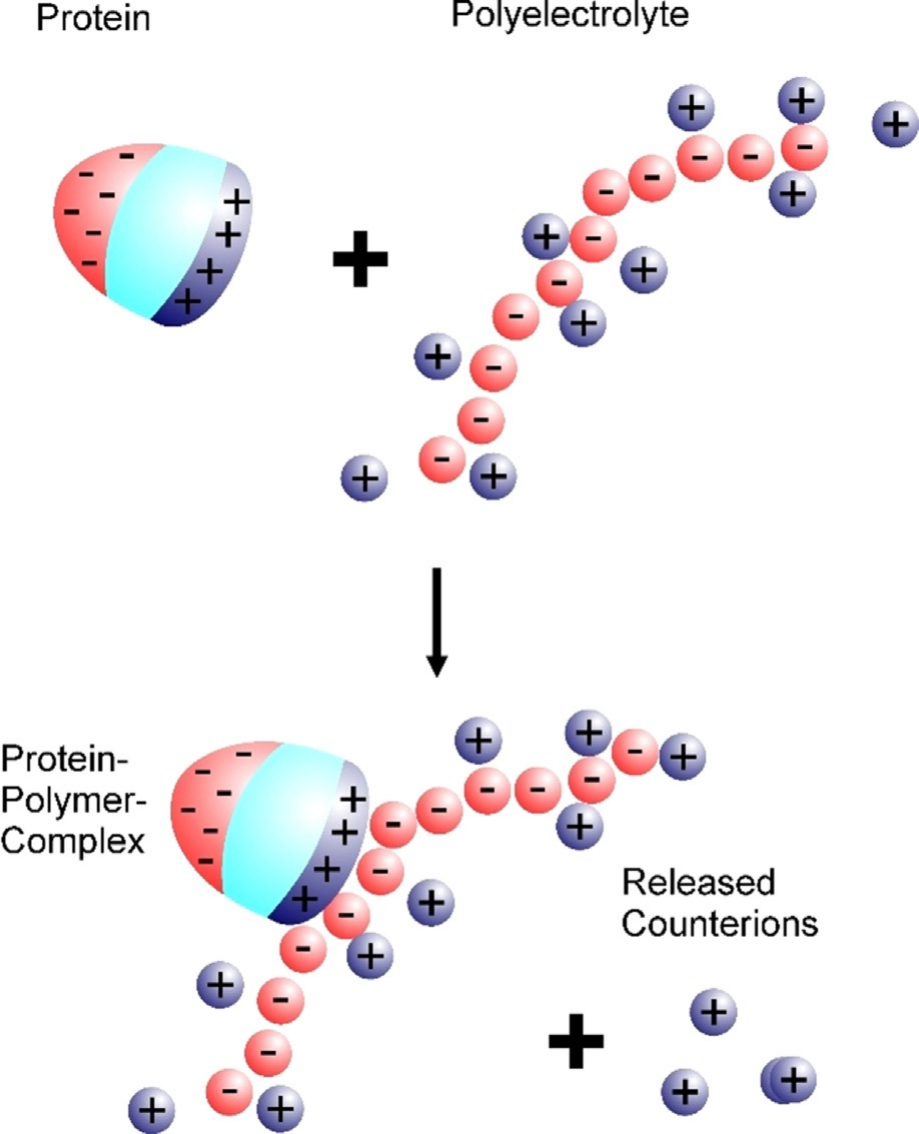
Interaction of proteins with highly charged polyelectrolytes, for example, DNA, by counterion release: Proteins bear negative (red) and positive charges (blue) on their surface. Above the isoelectric point, the overall surface charge is negative, but the positive patches remain. The polyelectrolyte bears a large number of charges that will lead to counterion condensation, that is, a certain fraction of the counterions are highly correlated with the polyelectrolyte, as shown here. Upon binding of the protein to the polyelectrolyte, a positive patch on the surface of the protein becomes a trivalent counterion of the polyelectrolyte. Thus, three counterions condensed on the polyelectrolyte are released upon binding. The free energy of binding will, therefore, be dominated by the entropic gain through the release of the counterions.[^12^, ^44^, ^45^] For the sake of clarity, only the condensed counterions are shown here. However, all the charges on the protein and the polyelectrolyte are balanced by an equal number of counterions.

To make progress in this field we need a quantitative understanding of the interaction of polyelectrolytes with proteins in general. In this Review we discuss recent work along these lines and how the analysis and the modeling of the interaction of polyelectrolytes with proteins can be used for a rational design of charged polymeric drugs. The central hypothesis of the present discussion is that this interaction is largely dominated by counterion release. Figure 1 shows this process in a schematic fashion: We consider the interaction of a protein carrying surface charges with a highly charged linear polyelectrolyte. A fraction of the counterions around the polyelectrolyte is “condensed”, that is, closely bound to the macroion.^[1]^ Proteins, in general, are polyampholytes, which carry patches of negative and positive charge on their surface. Most proteins bear an overall negative charge under physiological conditions. However, the patches bearing a positive charge remain and can interact with negatively charged polyelectrolytes such as DNA or heparin. In this way, the proteins become multivalent counterions of the polyelectrolyte, thereby releasing a concomitant number of its monovalent counterions. The gain in entropy thus achieved is the main driving force.^[2]^ Detailed considerations to be discussed further below demonstrate that this counterion release force is operative even under a physiological salt concentration of 150 mm.

Inspired by earlier work on the interaction of DNA with various proteins,[^2^, ^10^, ^13^, ^14^, ^46^, ^47^, ^48^, ^49^] we recently reconsidered the problem of counterion release by a series of thermodynamic studies related to the interaction of polyelectrolytes with proteins. First, the interaction of human serum albumin (HAS) with short‐chain poly(acrylic acid) in aqueous solution was studied by a combination of calorimetry and molecular dynamics simulations.^[50]^ We also studied the binding of dPGS of different generations^[51]^ to lysozyme[^45^, ^52^] and to HSA.^[53]^ Recently, this work has been continued to include a quantitative discussion of the role of water in the binding process.^[54]^ In addition to this, we demonstrated that MD simulations can reproduce the experimental binding constant of L‐selectin to second‐generation dPGS with surprising accuracy and be rationalized in terms of counterion release.^[45]^ The latter result could hence furnish a quantitative proof of earlier conjectures^[36]^ on the use of dPGS as an anti‐inflammatory drug. In this way we have acquired a rather advanced understanding of the interaction of dPGS with various systems of medical relevance.

**Figure 2:**
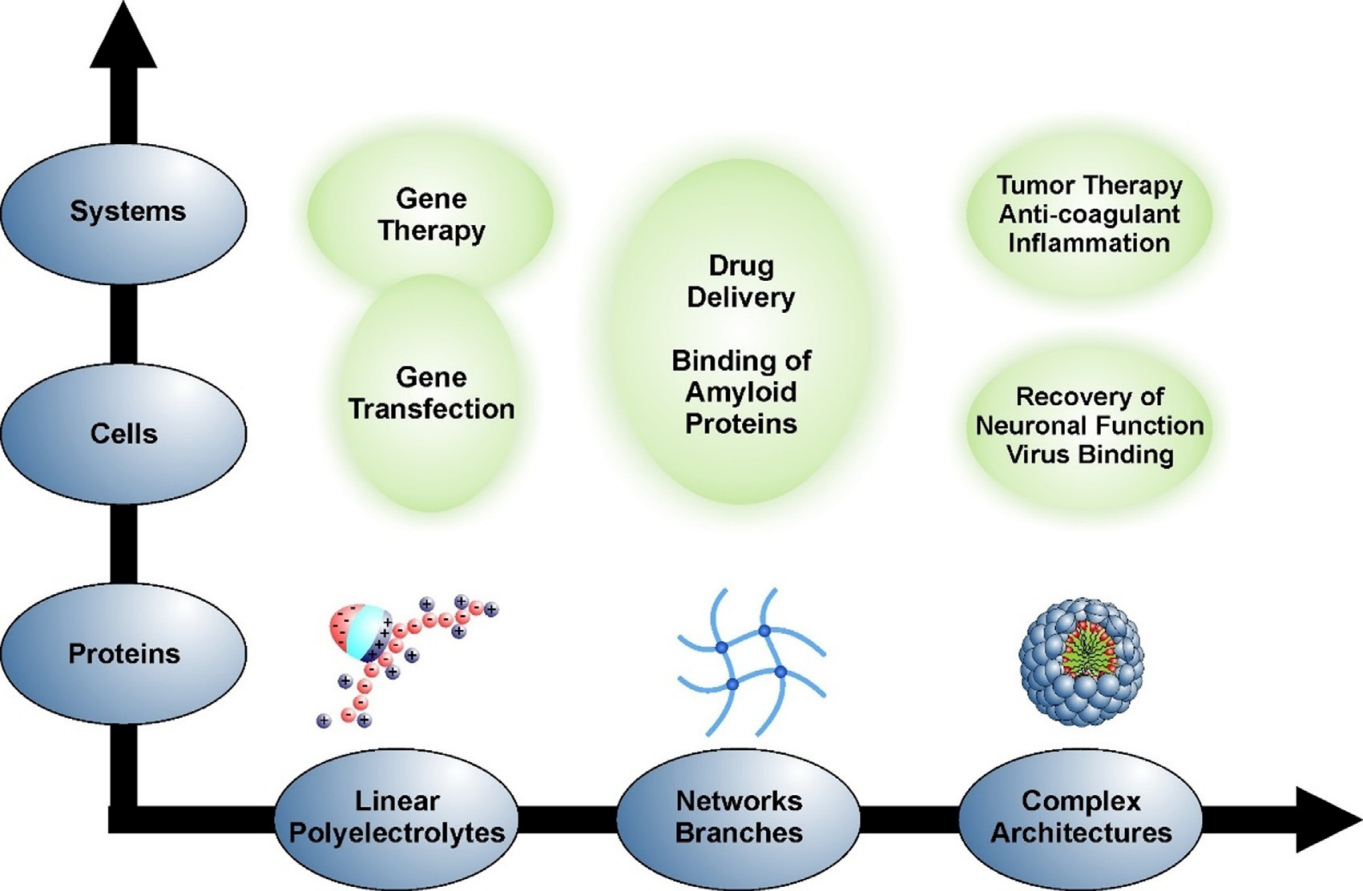
Interaction of polyelectrolytes with biosystems at different levels of complexity: Linear polyelectrolytes may be assembled into networks[^55^, ^56^] and branched systems. Ultimately, they may become building blocks for systems with higher complexity, for example, micelles with core–shell structures. Complexity on the biological side starts with single protein molecules that can interact with polyelectrolyte systems with various architectures. On this level, the therapeutic activity of polyelectrolytes can often be traced back to a blocking of proteins by a suitable polyelectrolyte system.[^31^, ^36^, ^39^, ^40^, ^42^, ^45^] Cells present the next level of complexity and their interaction with charged polymeric systems must be understood when considering these systems for, for example, drug delivery or gene transfection.[^20^, ^22^, ^57^, ^58^, ^59^] Organs present the highest level of complexity and the understanding of their interaction with synthetic polyelectrolyte systems is in its infancy. However, cationic polyelectrolytes with suitable architectures have recently been introduced as agents with anticoagulant reversal activity in blood.[^41^, ^42^, ^43^] The entire matrix of systems and problems gives a good overview of the possible medical problems to which synthetic polyelectrolytes may provide solutions.

Here we survey this work and how it can be applied for a better understanding and design of polyelectrolytes for medical purposes. Special emphasis will be laid on biomedical applications of dPGS and related systems. It is organized in terms of the matrix of chemical systems and biochemical problems with increasing complexity shown in Figure 2. Hence, the Review is subdivided in three parts as follows:

1. In the next section, we shall discuss the current understanding of the interaction of proteins with linear polyelectrolytes.[^2^, ^10^, ^11^, ^45^, ^49^, ^52^, ^53^] This section will survey the formation of complexes of proteins with DNA, which presents the best‐studied case in this field. At a higher level of biological complexity, problems related to drug delivery,[^32^, ^60^, ^61^] gene transfection, und ultimately gene therapy will be discussed. Glycosaminoglycans (GAGs) such as heparin or heparan sulfate, which consist of disaccharide units that may be sulfated, present another important class of natural, highly charged polyelectrolytes.[^18^, ^19^, ^62^] GAGs are important components of the extracellular matrix of cells. It has been recognized for a long time that the interaction with proteins is driven by electrostatic forces and counterion release.[^63^, ^64^, ^65^] This fact is underscored by more recent studies[^66^, ^67^, ^68^] and will be discussed as well.
2. The next level of complexity is given by dendritic and hyperbranched polyelectrolytes, charged networks, and polyelectrolyte brushes. Here, collective effects caused by the polymer architecture and their consequences for the interaction with proteins will be discussed. In particular, the counterion dPGS belongs in this section, which also contains the consequences for virus binding^[69]^ and inactivation as well as for the diagnostics and therapeutic use of dPGS in anti‐inflammation.[^36^, ^70^] This section will also highlight the interaction of proteins with polyelectrolytes of higher complexity, such as charged networks.[^21^, ^27^, ^31^, ^32^, ^37^, ^56^, ^71^] This work can be rationalized with recent theoretical studies on these systems.[^72^, ^73^, ^74^, ^75^, ^76^] The interactions of charged polyglycerols with cellular systems will also be discussed at this point.[^36^, ^77^, ^78^, ^79^, ^80^, ^81^]
3. Finally, a section devoted to complex polyelectrolyte architectures will highlight polyelectrolyte systems with higher complexity, such as micelles and designed polymeric structures that act as anticoagulants.[^25^, ^38^] These systems are far more difficult to understand in a quantitative fashion. However, there has been some progress towards medical applications recently, which will be discussed here. Thus, micelles coated by a layer of dPGS have been used as a drug in tumor targeting.^[38]^ Moreover, there have been recent successful developments of cationic polyelectrolyte drugs with heparin‐reversal activity in blood.[^41^, ^42^]

The entire discussion will highlight the general importance of electrostatic factors for the self‐assembly and biological activity of charged polymeric systems. As shown in Section 2, the driving forces are now rather well understood. Hence, this knowledge can now be used for the rational design and modeling of more complicated systems, as discussed in Sections 3 and 4. Steps in this direction will be discussed for all systems considered in Figure 2.

## 2 Fundamentals and Linear Polyelectrolytes

### 2.1 Theory

Up to now, the interaction of linear polyelectrolytes with proteins has been considered for two classes of problems, namely, the 1) interaction of natural polyelectrolytes (mainly DNA) with various proteins, and 2) interaction of synthetic polyelectrolytes with proteins. There is great number of studies devoted to the latter systems, since the classical investigations of Bungenberg de Jong in the 1930s (cf. the review of this work in Refs. [82, 83]). However, in many cases, mixing of synthetic polyelectrolytes with various proteins leads to phase separation (complex coacervates), which constitutes a problem of its own,[^82^, ^84^, ^85^, ^86^, ^87^] and which may lead to a complex phase behavior.[^88^, ^89^] DNA, on the other hand, forms well‐defined 1:1 complexes with proteins, such as polymerases,[^7^, ^90^, ^91^, ^92^, ^93^, ^94^] that can be understood in terms of a chemical equilibrium. This fact was recognized a long time ago[^2^, ^12^] and counterion release has been singled out as the main driving force for binding. The basic argument can be understood as follows: As depicted in Figure 1, there is a certain fraction of counterions that are condensed to the linear polyelectrolyte. The fraction of condensed counterions can be estimated from a relationship described by Manning:[^12^, ^95^] If *b* is the distance between two charges along the linear polyelectrolyte, a charge parameter *ξ* can be defined through Equation 1.

(1) $$\xi =\;\frac{\lambda_{B}}{b}$$

Here, *λ_B_* is the Bjerrum length ($\lambda_{B}=\frac{k_{B}T}{4\pi \epsilon \epsilon_{0}}$ ; *ϵ*: dielectric constant of the medium, *k_B_*: Boltzmann constant, *ϵ_0_*: permittivity of the vacuum, *T*: absolute temperature). If *ξ*>1, a fraction 1−1/*ξ* of the counterions will be condensed onto the linear chain, that is, strongly correlated with the polyelectrolyte. It is important to note that this fraction does not contribute to the osmotic pressure of the system. For DNA, this fraction amounts to about 70 % of all counterions. The condensed counterions can be regarded as a phase that may be characterized by a “surface concentration” *c_ci_*, which for DNA is of the order of 1 m.^[12]^

If we consider the interaction of such a highly charged polyelectrolyte with a protein, these condensed counterions must be treated as a reaction partner and, thus, contribute to the stoichiometry of the reaction.[^2^, ^12^] Hence, the complexation of a protein *P* with an anionic polyelectrolyte *PE* to a complex *PEP* is defined by Equation 2.^[2]^

(2) $$P+PE\vec{\leftarrow }PEP+\Delta n{}_{ci}M{}^{+}$$

Here, Δ*n_ci_* denotes the number of cations of type *M* ^*+*^ that have been released during the course of the binding reaction. The measured equilibrium constant *K_b_* can be formulated in terms of molar concentrations, and its relationship to the thermodynamic constant *K_T_* related to the activities of the components is given by Equation 3.^[2]^

(3) $$\ln K_{b}=\ln K_{T}+\ln\frac{\gamma_{P}\gamma_{PE}}{\gamma_{PEP}}-\Delta n_{ci}\ln\gamma_{M}-\Delta n_{ci}\ln\left[M^{+}\right]$$

Here, *γ_P_, γ_PE_*, *γ_PEP_*, and *γ_M_* denote the activity coefficients of the protein, the polyelectrolyte, the complex, and the free ions, respectively (see also the discussion of this problem in Ref. [54]). Since the concentration *[M^+^]* of monovalent cations is much larger than the concentrations of the polyelectrolyte and the protein, *[M^+^]* equals, to an excellent approximation, the concentration of added salt *c_s_*. First, the activity coefficient of the ions can be disregarded, since we deal mostly with small concentrations of the ions. Moreover, it can be shown that the second term on the right‐hand side of Equation (3) related to the activity coefficients give a small but non‐negligible contribution for linear polyelectrolytes that scales with ln(*c_s_*).[^44^, ^96^] This term contains the Debye–Hückel interactions of the various parts of the complex. For complexes of proteins with spherical polyelectrolytes, all contributions from activity coefficients may be shown to be small and negligible to a first approximation.^[54]^ Hence, to a good approximation, Equation (3) can be simplified to Equation 3a).

(3a) $$\ln K_{b}\approx \ln K_{b}\left(1M\right)-\Delta n_{ci}\ln c_{s}$$

Here, *K_b_*(1 m) is the binding constant extrapolated to one molar salt concentration. Thus, the stoichiometric coefficient Δ*n_ci_* is given to a good approximation by $-d\ln K_{b}/d\ln c_{s}$ , that is, by the negative slope of the plots of the log of the measured equilibrium constant *K_b_* against log *c_s_*. Only at very low ion concentrations of the order of 1 mm and less will the data deviate from linearity because of a non‐negligible repulsive Debye–Hückel interaction.[^54^, ^97^]

Many years ago, Tanford argued that Equation (3) needs to be supplemented by a term that takes into account the number Δ*w* of released or bound water molecules during the course of complex formation.^[98]^ Thus, a term scaling such as −*(n_i_/n_w_)*Δ*w* should be included in Equation (3). Here, *n_i_* and *n_w_* denote the molar number of ions and of water molecules in the system, respectively. However, *n_i_* is typically of the order of 10^−2^ to 10^−1^, whereas *n_w_* is 55.6. Hence, this term, which reflects the change of hydration during complex formation, is small and can be dismissed for low ion concentrations *n_i_*.[^2^, ^98^] This term comes into play for high ion concentrations in excess of 1 m.[^99^, ^100^, ^101^] In this case, plots of log *K_b_* versus log c_s_ are no longer linear. This problem has been studied in a series of important investigations by Bergqvist, Ladbury, and co‐workers.[^99^, ^100^, ^102^, ^103^] Here, plots according to Equation (3) indeed exhibit a marked curvature, which can be explained in terms of a model taking into account the release of water molecules during binding.[^98^, ^104^, ^105^] A much‐refined discussion of the release of water was presented by Record and co‐workers,[^106^, ^107^] who demonstrated that Δ*w* is intimately related to the preferential adsorption of the ions on the surface of the biomolecule (cf. Ref. [106] and further references therein). The model of vander Meulen et al.^[107]^ predicts that Δ*w* vanishes if there is no preferential adsorption of the co‐ or counterions. The analysis of experimental data on the binding of proteins to DNA led vander Meulen et al. to the conclusion that Δ*w* is small if salts in the middle of the Hofmeister series,^[108]^ for example, NaCl or KCl, are used. Hence, Δ*w* will be small, and nearly all complexes of DNA with proteins have been modeled by Equation (3a).[^2^, ^10^, ^13^, ^14^, ^46^, ^47^, ^48^, ^49^]

Equation (3a) can be used to analyze the measured binding constant *K_b_* further by splitting it up through extrapolation to a 1 m salt concentration. Thus, *K_b_* now consists of a reference part *K_b_*(1 m) and a term depending solely on the release of counterions.[^10^, ^11^] Equation 4 gives Equation 5, where Δ*G_res_* is the residual of the Gibbs free energy of binding derived from *K_b_*(1 m), whereas Δ*G_ci_* denotes the part related to counterion release.^[11]^

(4) $$\Delta G_{b}=-RTln\;K_{b}$$

(5) $$\Delta G_{b}=\Delta G_{res}+\;\Delta G_{ci}\;$$

Extrapolation of the measured *K_b_* value to a 1 m salt concentration according to Equation (5) leads to Δ*G_res_*, that is, Δ*G_res_*=−*RT*ln(*K_b_*(1 m)), and in turn to Δ*G_ci_*. Here, the quantity Δ*G_res_* denotes all contributions to the free energy of binding which are not from counterion release, such as direct electrostatic interaction,^[54]^ hydrogen bonding, or salt bridges as well as other effects. In this way, the salt concentration of 1 m constitutes a reference state.

From the above approximations, counterion release is a fully entropic effect and we obtain Equation 6.^[54]^

(6) $$\Delta G_{ci}≅-T\Delta S_{ci}≅RT\Delta n_{\mathrm{ci}}\ln\frac{c_{ci}}{c_{s}}$$

Here, Δ*S_ci_* denotes change in entropy of the counterions, which can be calculated from the surface concentration *c_ci_* introduced above.[^45^, ^52^, ^109^, ^110^, ^111^] The quantity *c_ci_* can be estimated for linear polyelectrolytes from *ξ* as prescribed by Manning^[12]^ or it can be deduced from molecular dynamics simulations, as shown recently.^[45]^ Moreover, with the total binding entropy Δ*S_b_* being known, the residual part Δ*S_res_* can be calculated with Equation 7.[^11^, ^54^]

(7) $$\Delta S{}_{\mathrm{res}}\left(T\right)=\Delta S{}_{b}\left(T\right)-\Delta S{}_{\mathrm{ci}}$$

It is evident from Equations (1)–(7) that a comprehensive thermodynamic analysis of the binding of proteins to polyelectrolytes can be achieved.

We now turn to an important tool that has been pivotal for thermodynamic analysis: In the last two decades isothermal titration calorimetry (ITC) has become the central tool for the analysis of complex formation in natural and synthetic systems.[^112^, ^113^] ITC measures directly the heat evolved upon complex formation with high precision. Thus, by using ITC, the *K_b_* value of DNA with a great variety of proteins can be determined with high accuracy;[^112^, ^114^, ^115^] a large number of such studies have now been carried out.[^13^, ^47^, ^90^, ^91^, ^116^, ^117^, ^118^, ^119^, ^120^, ^121^, ^122^, ^123^] It is fair to state that most of the quantitative knowledge on the interaction of polyelectrolytes with proteins has been acquired by ITC experiments and this method holds great promise for further understanding of these systems, in particular when applied to the design of pharmaceutical systems.[^112^, ^124^]

### 2.2 Enthalpy–Entropy Compensation

Investigations by ITC and application of Equation (4) have led to a great amount of precise thermodynamic data. Here, studies of the dependence of the binding constant *K_b_* on temperature revealed a strong enthalpy–entropy compensation, that is, most of the measured binding enthalpy is balanced by a concomitant entropic contribution. This enthalpy–entropy compensation (EEC)[^13^, ^49^, ^125^, ^126^, ^127^, ^128^] has been a controversial subject for quite some time.[^129^, ^130^, ^131^, ^132^] However, Grunwald and Steel^[133]^ pointed out many years ago that the EEC is the natural consequence of the rearrangement of solvent molecules around a solute. This idea was reviewed carefully more recently by Liu and Guo.^[134]^ Moreover, Li et al. showed that the EEC is a real effect with a sound experimental basis.^[135]^ This is in full agreement with recent experimental studies of the Whitesides group that explain the EEC by the reformation of the water network around the complex.^[132]^ Jen‐Jacobson and co‐workers showed that EEC exists in systems of biological relevance.[^13^, ^14^, ^49^, ^136^] Synthetic systems have been studied with equal intensity,^[134]^ and from the vast amount of literature we only cite the very recent investigation by Schönbeck and Holm^[137]^ on the EEC for complexes of cyclodextrin with various host molecules. Dragan et al. have recently suggested that EEC may be related to the release or uptake of water.^[11]^ In Section 3 we will discuss our recent studies on the EEC for the interaction of dPGS with various proteins, which come to the same conclusions.[^52^, ^53^, ^54^] Summing up this survey of theoretical and experimental work that now extends over 50 years,^[125]^ it is clear that EEC is a ubiquitous phenomenon that has been firmly established by a great number of experimental studies.

### 2.3 Interaction of DNA with Proteins

In this section, the above conclusion will be compared with experimental findings. Here we start with a survey on studies carried out on natural systems. For a long time^[2]^ the interaction of DNA with various proteins has been analyzed in terms of Equation (3). The application of Equation (3a) to the formation of protein/DNA complexes has been analyzed for a wide variety of systems by Privalov, Dragan, and Crane‐Robinson.^[10]^ In all cases, straight lines were obtained indeed by application of Equation (3a). Moreover, the number of released ions Δ*n_ci_* is found to be strictly correlated to the number of ionic contacts seen between DNA and the protein in crystal structures (cf. the discussion in Ref. [10]). It should be noted that ion‐specific effects may change Δ*n_ci_* slightly and should be considered carefully.^[10]^ Furthermore, the binding of DNA to proteins may lead to changes of the secondary structure and the partial refolding of proteins. This point has been discussed in detail by Privalov et al.[^10^, ^138^] and by Jen‐Jacobson et al.[^13^, ^49^]

Dragan et al.^[11]^ have used Equation (5) to split the measured Δ*G_b_* into the part corresponding to counterion release and a residual part. A similar analysis has been applied to the binding of DNA to proteins by other research groups as well. In particular, Dragan et al. could demonstrate that the EEC is an entirely non‐electrostatic phenomenon: Plotting the measured binding enthalpy Δ*H_b_* against the residual entropy Δ*S_res_* resulted in a perfect master curve^[11]^ for some 30 DNA/protein complexes. The authors concluded that the EEC observed in these systems must, hence, be due to hydration, that is, the release or uptake of water. The same master curve was found by us for the system dPGS/lysozyme,^[54]^ which will be discussed further in Section 3.

### 2.4 Interaction of RNA with Proteins

There are much fewer thermodynamic studies on the interaction of RNA with various proteins that consider explicitly the dependence on ionic strength in terms of Equation (3). Maiti and co‐workers presented a comprehensive study of the interaction of HIV‐1 TAR RNA and Tat‐derived arginine‐rich peptides by various techniques, including ITC.^[139]^ Plots of the binding constant according to Equation (3a) are linear and show that one ion is released upon binding. Samatanga et al. investigated the interaction of single‐stranded RNA with various proteins containing various RNA‐recognition motifs by ITC.^[140]^ Use of Equation (3a) demonstrated that the ionic interaction is small for these systems and Δ*G*_res_ is mainly dominated by hydrogen bonding. It is interesting that both studies found the interaction of RNA with the respective proteins to be mainly driving by enthalpy. Cababie et al. recently presented a carefully conducted thermodynamic study of the interaction of the NS3 helicase with single‐stranded RNA by using fluorescence titration.^[141]^ Equation (3a) was shown to give a good description of the measured binding constants. Typically, Δ*n_ci_* was found to be five and rather independent of the ions used for adjusting *c_s_*.

### 2.5 Glycosaminoglycans (GAG) as Highly Charged Polyelectrolytes

**Figure 3:**
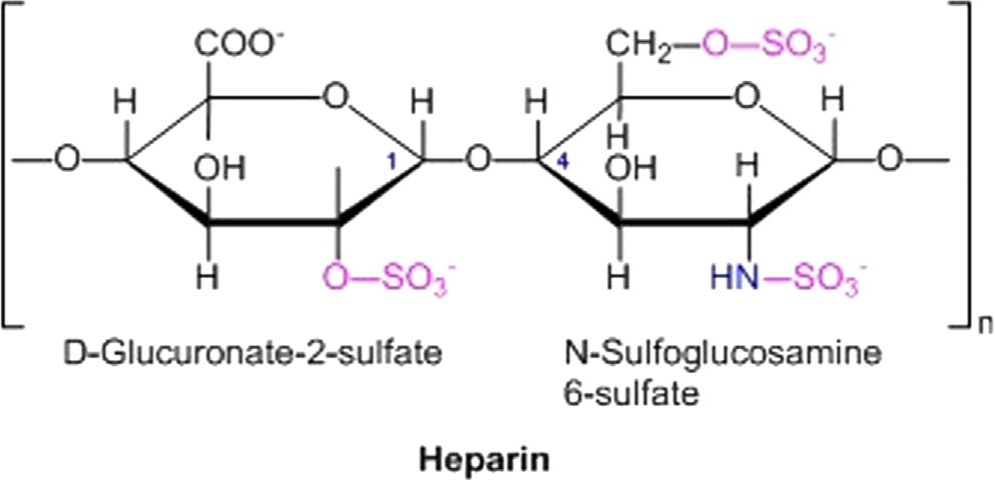
Chemical structure of the monomer unit of heparin.

Glycosaminoglycans (GAGs) such as heparin consist of oligosaccharide units that may be sulfated.[^16^, ^18^, ^19^] Animal tissues contain multiple sulfated glycosaminoglycans, such as heparan sulfate (HS), heparin, chondroitin sulfate (CS), dermatan sulfate (DS), and keratan sulfate (KS), which can be distinguished by their sugar constituents and sulfation pattern.^[19]^ Figure 3 displays the repeating unit of heparin, which is the most‐studied GAG. In general, GAGs exhibit variations of the molecular structure, and the degree of sulfation may change. Thus, Figure 3 shows only the most abundant repeating unit (see the discussion of this point in Ref. [19]). Heparin has four charges per disaccharide repeat unit and is one of the most highly charged biopolymers. Heparin acts as an anticoagulant^[42]^ and can interact with various proteins.[^17^, ^62^, ^142^, ^143^] Moreover, hydrogels consisting of heparin and modified GAG units are capable of sequestering proteins and, in particular, cytokines that may prevent wound healing.[^30^, ^56^, ^144^] Heparan sulfate (HS), which is slightly less sulfated than heparin, is located in the extracellular matrix and serves as a primary receptor for many pathogens, such as bacteria and viruses. HS was shown to be involved in the infection by many viruses through facilitating their internalization or interaction with secondary receptors.[^37^, ^69^, ^79^, ^80^, ^145^] Thus, it is now clear that attachment of many viruses to cells involves electrostatic interactions with HS.^[145]^ Therefore, a number of sulfated molecules have been investigated as inhibitors (cf. the discussion of Table 2 in Ref. [145]). In general, GAGs have been tested for sequestering or the defined delivery of cytokines and growth factors.^[19]^ A thorough and quantitative understanding of the interaction of GAGs with proteins is a central problem in biomedical research.

Since the early work of Olson et al.^[63]^ and of Mascotti and Lohman^[64]^ it is well‐established that electrostatic interaction and counterion release play a central role in the binding of proteins to heparin.[^83^, ^146^, ^147^, ^148^, ^149^, ^150^, ^151^, ^152^] The prevalence of electrostatic interactions beween proteins and heparin has been corroborated by a considerable number of investigations;[^68^, ^83^, ^151^, ^153^, ^154^] a survey of the older literature may be found in the review by Seyrek and Dubin from 2010.^[65]^ Thus, linear plots of log *K_b_* versus log *c_s_* are found in a number of investigations.^[65]^ The number of quantitative studies employing Equation (3), however, is rather small given the obvious importance of GAGs as biomaterials.[^19^, ^155^, ^156^] It is important to note that electrostatic interactions with heparin are already used in medical applications. Thus, protamine, which is a highly cationic polypeptide, is used to neutralize an overdose of heparin.^[157]^ A detailed discussion of this application will be given in Section 4.

### 2.6 Linear Synthetic Polyelectrolytes that Interact with Proteins

Less quantitative work has been carried out using ITC on the interaction of synthetic linear polyelectrolytes with proteins. Careful work by Dubin and co‐workers, however, has shown that charge–charge interactions are central for the understanding of the complex formation between proteins and various polyelectrolytes.[^83^, ^158^, ^159^, ^160^, ^161^, ^162^] A first investigation of the linear polyelectrolyte poly(allylamine hydrochloride) with BSA by Ball et al.^[163]^ using ITC demonstrated that the driving force for complex formation is entropic. Equation (3a) was used repeatedly to model the interaction, and in many cases a good linearity was found, at least at higher ionic strength (see the discussion of this problem in Ref. [109]) Recently, a careful investigation of this problem was presented by Lounis et al.,[^164^, ^165^] who demonstrated that Equation (3a) provides an excellent description of experimental data for the interaction of linear and dendrigraft poly(lysine) with synthetic anionic polyelectrolytes.

**Figure 4:**
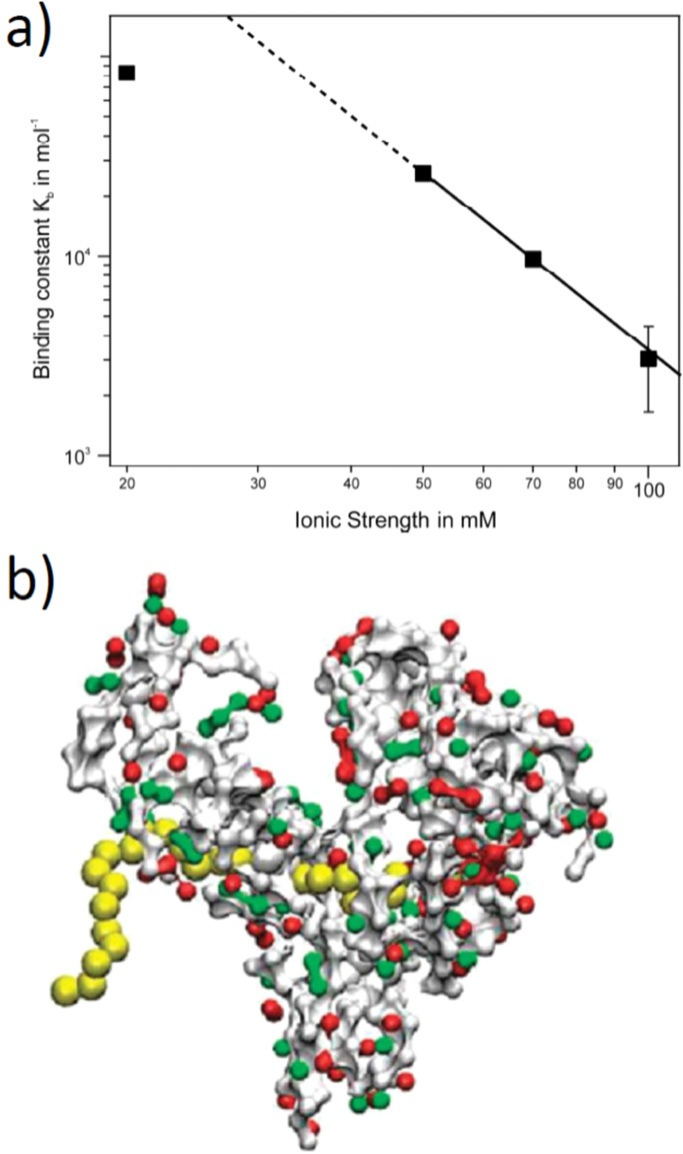
Interaction of linear synthetic polyelectrolytes with proteins. a) The binding constant of poly(acrylic acid) to human serum albumin (HSA) is plotted against the log of the salt concentration. At high salt concentrations, there is a linear relationship, the slope of which gives the number of released counterions according to Equation (3). Deviations at low salt concentrations point to a residual Debye–Hückel repulsion between the protein and the polyelectrolyte. b) MD simulation of the interaction of the interaction of HSA with poly(acrylic acid). The polyelectrolyte is bound to the Sudlow II site of HSA.^[50]^

We have recently analyzed the interaction of human serum albumin (HSA) with short chains of poly(acrylic acid) (PAA) in aqueous solution as a function of the ionic strength and temperature.^[50]^ The low molecular weight of PAA prevented the formation of complex coacervates, and ITC could be used for a fully quantitative analysis of Δ*G_b_*. Figure 4 shows that Equation (3) is valid for higher ionic strengths, whereas low salt concentrations led to deviations, as discussed above. The simplicity of this systems allowed us to perform molecular dynamics (MD) simulations of this binding process. Figure 4 b displays a simulation snapshot of a complex between HSA and poly(acrylic acid). The simulations suggest that the linear polyelectrolyte is bound in the Sudlow II site, which is to be expected from earlier studies of HSA. Moreover, the number of released counterions could be obtained from the simulations. This number can be compared to the experimental result obtained through application of Equation (3a) (Figure 4 a). We found three counterions to be released in the binding process from simulations as well as from the experiment.^[50]^ Thus, the good agreement between theory and experiment corroborates the analysis of binding in terms of Equation (3a).^[50]^

It is important to check whether a given protein is changed upon interacting with a polyelectrolyte. In this case, a part of the caloric signal would be due to a partial denaturation or a refolding of the protein. For the case of DNA interacting with various proteins, this problem has been investigated by Privalov et al.^[138]^ (see also the discussion in Ref. [11]) and by Jen‐Jacobson et al.^[13]^ For the system HSA/PAA discussed above, the resulting complexes have been analyzed by small‐angle neutron scattering (SANS).^[166]^ No significant changes in the overall structure of HSA could be detected by this method. CD spectroscopy is an excellent tool to reveal possible changes in the secondary structure of complexes.[^167^, ^168^, ^169^, ^170^] Thus, if HSA interacts with dendrimers having partially hydrophobic moieties, there is a significant loss of α‐helices.^[170]^ Tests on the secondary structure of a protein in a complex with a given polyelectrolyte are therefore mandatory.

From the results obtained for a large number of natural and synthetic systems, one can state that the analysis of Δ*G_b_* in terms of Equation (3) has led to a semiquantitative understanding of the interaction of polyelectrolytes with proteins. The effect of counterion release can be separated from the other factors by use of Equation (3a), which provides the first step towards the quantitative understanding of Δ*G_b_*. ITC measurements have turned out to be central for these studies and MD simulations will allow us to acquire a molecular understanding of the thermodynamic data.

### 2.7 Biotechnological and Medical Applications of Linear Synthetic Polyelectrolytes

An important application of charge–charge interaction is gene delivery by nonviral vectors.[^22^, ^23^, ^171^, ^172^, ^173^] Here, cationic polyelectrolytes are used to compact DNA and RNA by formation of so‐called polyplexes.^[20]^ The micelles and aggregates formed by this interaction may then form more complex supramolecular structures.^[174]^ Polyethyleneimine (PEI) has been the cationic polyelectrolyte of choice.[^175^, ^176^] Concerns about the inherent toxicity of PEI has led to an enormous number of studies that have tried to improve gene delivery by designed block copolymers, which has recently been review by Kataoka and co‐workers^[25]^ and by Reineke and co‐workers.^[177]^ Charged dendrimers have also been used for this purpose.^[178]^ An interesting application is the delivery of proteins through a suitable packaging by block copolymers with charged blocks. Here, we only cite recent work on block copolymers that deliver the CRISPR/Cas9 system^[179]^ and the nanoformulation of the brain‐derived neurotrophic factor (BDNF) by a block copolymer containing a poly(glutamic acid) block.^[60]^ In the latter case, cationic patches on the BDNF interact electrostatically with the negatively charged block, and the resulting supramolecular structures then lead to a better delivery of the BDNF.

It is interesting to note that linear polyelectrolytes may act as synthetic chaperones thus, guiding proteins to adopt the correct tertiary structure. This was shown by Semenyuk et al. in a series of careful studies.[^180^, ^181^, ^182^] The complexes of the proteins with polyelectrolytes such as polystyrene sulfonic acid also stabilized the structure of the proteins against aggregation in a very efficient manner. Furthermore, the complexes were stable at temperatures where the free protein would be denatured. This complexation of proteins with linear polyelectrolytes hence holds the promise for further biotechnological applications.

A totally different problem of medical relevance arises when considering the interaction of short‐chain polyelectrolytes and small charged molecules such as phenylacetic acid with HSA. These substances adhere strongly to HSA and are, therefore, difficult to remove by a conventional dialysis. Patients with chronic kidney disease have high concentrations of such uremic toxins, which may lead to a higher cardiovascular morbidity.[^166^, ^183^, ^184^] ITC is a central tool for analyzing the interaction of such toxins with HSA.^[166]^ Here, short polyelectrolytes may serve as models for the so‐called middle molecules that present uremic toxins stemming from degraded proteins (cf. Ref. [166]). The interaction with HSA is mainly depends on counterion release, as shown above.^[50]^ Small toxins such as phenylacetic acid or indoxyl sulfate, however, interact mainly with the hydrophobic sites of HSA and exhibit a rather high binding constant.^[166]^ Removal of uremic toxins is, hence, a central task of clinical nephrology and an improved thermodynamic understanding of their interaction with HSA is absolutely necessary.

## 3 Charged Networks, Dendritic and Hyperbranched Polyelectrolytes, and Polyelectrolyte Brushes

### 3.1 Dendritic and Hyperbranched Polyelectrolytes

The previous section has demonstrated that the interaction of proteins with linear polyelectrolytes can be largely understood and modeled. In a next step, we consider more complicated structures and start with branched and dendritic polyelectrolytes. Highly charged dendrimers have been the subject of intense research since the first pioneering theoretical study by Welch and Muthukumar^[186]^ in 1998. Charged dendrimers have been studied for gene transfection for a long time,[^172^, ^178^, ^187^, ^188^, ^189^, ^190^, ^191^] and discussed for drug delivery in general.[^192^, ^193^, ^194^]

**Figure 5:**
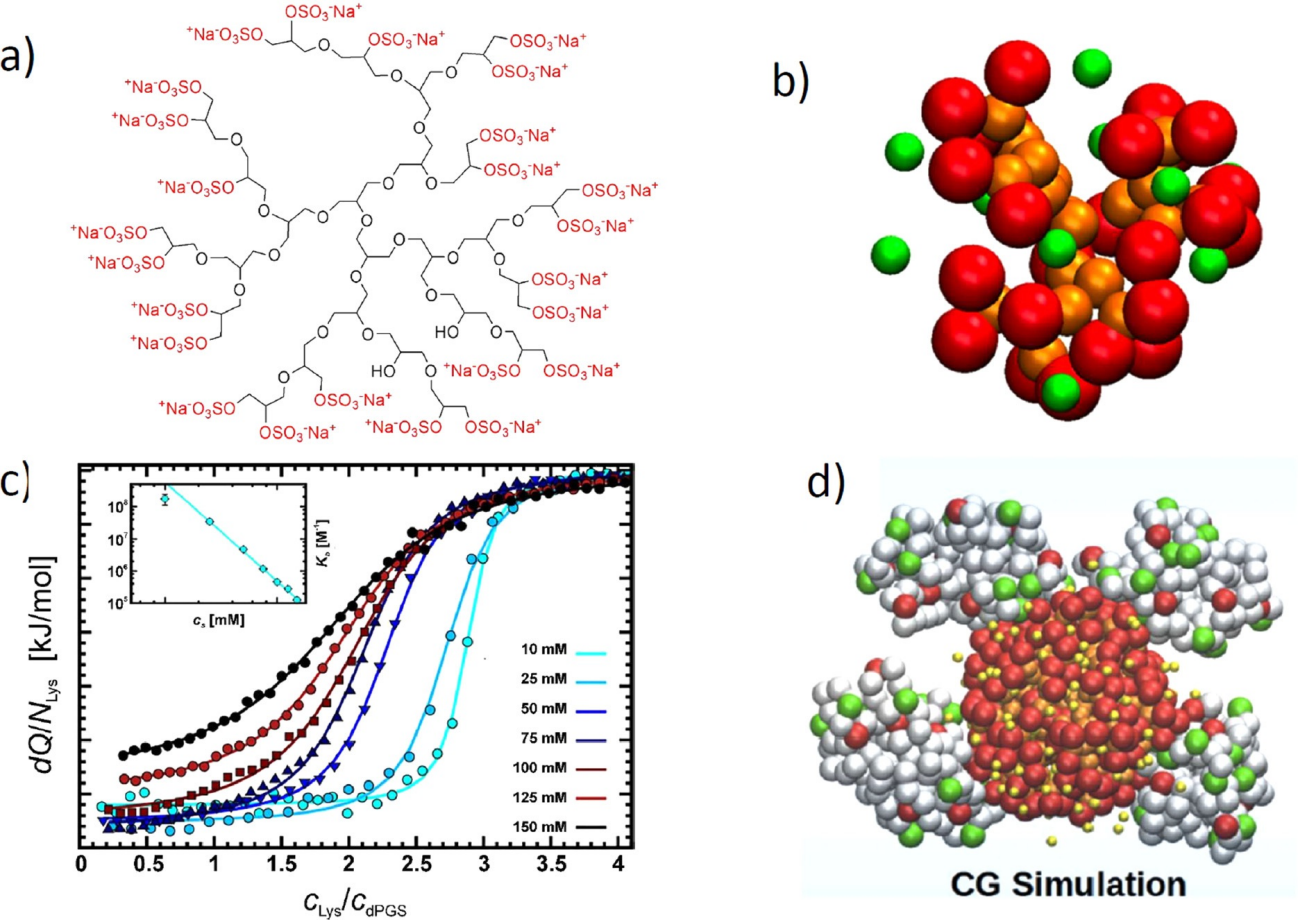
Sulfated dendritic polyglycerol (dPGS) and its interaction with proteins. a) Chemical structure of dPGS. The scaffold consists of a highly hydrophilic dendritic or hyperbranched structure, with each end group carrying a sulfate group.^[185]^ b) Snapshot of the coarse‐grained structure of a second‐generation dPGS. Red beads mark the terminal sulfate groups of the dendritic structure, and yellow beads mark its scaffold. The counterions are displayed as green beads.^[51]^ c) Interaction of a second‐generation dPGS with lysozyme measured by ITC at different ionic strengths. The incremental heat per injection is plotted against the molar ratio of lysozyme to dPGS in aqueous solution. The inset displays the log of the resulting binding constant as a function of the log of the salt concentration according to Equation (3).^[45]^ d) Coarse‐grained MD simulations of the interaction of a third‐generation dPGS with lysozyme. The snapshot shows a complex of the central dPGS molecule with four lysozymes.^[45]^

We have recently investigated charged dendritic polyglycerols. Figure 5 gives a survey of these systems and the main results achieved so far. Figure 5 a displays the chemical structure of the polyanionic dendritic polyglycerol sulfate (dPGS). The scaffold consists of the highly hydrophilic polyglycerol, on to which sulfate groups are appended. These systems based on hyperbranched polyglycerol were made for the first time in 2004^[185]^ and used for various medical purposes.^[34]^

In a first step, for a better understanding of the interaction of dPGS with proteins and more complicated biological systems (see Figure 2), we have studied the spatial structure of these dendrimers. First, MD simulations were used to explore the interaction of the highly charged systems with their counterions.^[51]^ Figure 5 b displays a typical simulation snapshot of a dPGS dendrimer of the 2nd generation. The segments of the scaffold and the end groups were modeled in a coarse‐grained fashion. It is clear that these systems present rather dense structures, where the charged groups are located mainly at the outside. Simulation can serve to define an approximate surface of the dendritic structure that may be compared with measured hydrodynamic radii.^[51]^ The counterions are highly correlated to the macroion and form a dense layer on the surface of the dendrimer. Hence, a surface concentration *c_ci_* may be defined in the same way as already discussed in conjunction with linear polyelectrolytes [see the discussion of Equation (1)]. This surface concentration is on the order of 1 m for a dPGS of second generation and rises considerably for higher generations.

It is important to understand that correlation of the counterions with the highly charged dendrimer proceed on a mesoscopic level, in which molecular details play a minor role. Coarse‐grained simulations may hence lead to a better understanding of the counterion release mechanism, but cannot reveal details of interactions related to, for example, hydrogen bonding. However, MD simulations can be directly compared to the hydrodynamic radius, and the effective charge determined experimentally.^[51]^ These data agree with the simulations within the limits of error. In particular, the effective surface charge levels off with increasing number of generations, while the bare charge increases exponentially. Thus, these systems exhibit the charge renormalization expected for highly charged spherical macroions.^[51]^ This charge renormalization must be kept in mind when comparing the interaction of charged dendrimers of different generations with proteins. Evidently, these systems are expected to interact with proteins through counterion release in the same way as already discussed for the linear systems above. Furthermore, a salt concentration of 1 m will lead to a vanishing contribution of the counterion release [see Eq. (6)] and provide a good reference state. The results of these coarse‐grained simulations have been checked and fully corroborated by atomistic simulations with explicit water.^[195]^

In a second step, MD simulations turned out to be highly revealing when studying the interaction of dPGS with proteins.[^45^, ^53^] Figure 5 c displays the ITC diagrams for the interaction of a second‐generation dPGS with lysozyme in aqueous solution. A parameter of the different curves is the ionic strength in these solutions, which ranges from 10 mm to a physiological concentration of 150 mm. The weakening of the interaction with increasing ionic strength is directly apparent, and the inset of Figure 5 c shows that the logarithm of the binding constant scales linearly with the salt concentration *c_s_* in solution, as predicted by Equation (6). The slope of these lines leads directly to the number of released counterions (three), in good approximation to that already discussed in conjunction with Equation (6). Moreover, the free energy of binding Δ*G_b_* is nearly independent of temperature, which is followed by a strong compensation of the enthalpy and the entropy of binding. This particular point will be discussed further in Section 3.5.

MD simulations now lead to data that can be directly compared to experiments: Figure 5 d displays a typical snapshot of a complex in which four lysozyme molecules are bound to a third‐generation PGS. First of all, the interaction of the protein with a dPGS molecule is quantitatively obtained by steered Langevin simulations: Here the centers of gravity of the dPGS and the protein are kept at a fixed distance and the force between the two molecules is averaged. By integration over the distance, we obtain a potential of the mean force, the maximum of which is the free energy of binding Δ*G_b_*. Moreover, the number of released counterions and the average number of bound proteins can directly be obtained from these simulations and compared to experiments. A comparison with experimental data showed an excellent agreement.[^45^, ^50^, ^53^] The number of released counterions derived from the simulations compare very well with the experimental data (cf. the discussion of Figure 5 c). Moreover, it was demonstrated that the free energies derived from simulations can be directly compared to experimental data. Here again, good agreement is found.^[45]^ Hence, MD simulations provide an excellent tool for the quantitative understanding of the interaction of polyelectrolytes with proteins.

The same combination of ITC and MD simulations was recently applied to complexes formed between second‐generation dPGS and HSA.^[53]^ The same features as discussed for the dPGS/lysozyme system are found here as well: A well‐defined 1:1 complex is formed and counterion release is found to be the main driving force. Again, a strong EEC is found by ITC measurements as a function of temperature. The experimental binding constant again agrees with the simulated one within the limits of error. The CD spectra of the complex measured up to 310 K showed no significant change when compared to the spectra recorded at room temperature. This finding is in contrast to complexes formed by a PAMAM dendrimer with HAS, where a major reduction of the α‐helix content was found because of partial unfolding.^[170]^

**Figure 6:**
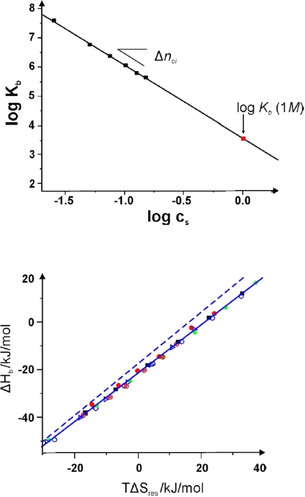
Thermodynamic analysis of the binding of lysozyme to a second‐generation dPGS.^[54]^ Top: A plot of log *K_b_* versus log *c*_s_ as suggested by Equations (4) and (6). There is a perfectly linear relation in this double‐logarithmic plot, in which the slope gives the number of released counterions, as discussed in conjunction with Equation (3a). The linear relationship is used to extrapolate the binding constant Δ*G_b_* at a salt concentration of 1 m. *K_b_*(1 m) is related to Δ*G_res_*, the residual of the Gibbs free energy of binding according to Equation (5,) and reflects all contributions to Δ*G*_b_ not related to counterion release. Bottom: Enthalpy–entropy compensation for the data obtained on the system dPGS‐G2/lysozyme. The enthalpy Δ*H_b_* is plotted against *T*Δ*S_res_*=*T*Δ*S_b_*−*T*Δ*S_ci_* according to Equation (7). The solid line denotes the fit by Equation (8). The dashed line shows the master curve derived by Dragan et al.^[11]^ for a wide variety of systems in which DNA interacts with proteins.

The MD simulations of dPGS interacting with proteins[^45^, ^52^, ^53^] were carried out using only implicit water, that is, all simulations have assumed water to be a structureless medium with a given dielectric constant. Here the question arises in what way water is involved in the process of binding. This problem has recently been elucidated further by reconsidering the measured binding constant of the dPGS/lysozyme^[52]^ system in terms of Equations (5)–(7).^[54]^ Figure 6 a shows a typical plot of the measured binding constant as a function of the salt concentration *c_s_* according to Equation (3a). The strict linearity of this plot allows us to determine the number of released counterions with good accuracy. Moreover, the binding constant *K_b_*(1 m) could be extrapolated with equal precision and used for the breakdown of the measured data, according to Equation (5), into to a part (Δ*G_ci_*) solely due to counterion release and a residual part (Δ*G_res_*) due to specific interactions, such as salt bridges and hydrogen bonding. At the same time, the enthalpy of binding was largely balanced by an entropic term of comparable magnitude. Figure 6 b displays a plot of the enthalpy of binding versus the residual entropy of binding Δ*S_res_* multiplied by *T* [see the discussion of Eq. (7)]. All data collapse on a single master curve that shows that the breakdown of the free energies of binding according to Equation (5) provides an excellent approximation for the data. This master curve is given by Equation 8.

(8) $$\Delta H_{b}=-21.3+1.017\cdot T\Delta S_{res}$$

The intercept of −21.3 kJ mol^−1^ is, hence, the average value of Δ*G_res_* for the present system, and the slope very near to unity shows that there is a nearly full compensation of the enthalpy by entropy.

It is interesting to note that this master curve obtained for the dPGS/lysozyme^[52]^ system shown in Figure 6 b) virtually coincides with the master curve found by Dragan et al. for some 30 systems in which DNA interacts with various proteins (dashed line in Figure 6 b). The slope of this master curve is slightly higher than 1 (1.09 vs. 1.017 for the dPGS/lysozyme system) and Δ*G_res_* is slightly smaller. Despite these small differences, both investigations agree that the binding of proteins to DNA leads to a marked EEC with a non‐zero value of Δ*G_res_*. Dragan et al.^[11]^ explained the marked EEC by the uptake or release of water during binding. Our findings^[54]^ underscore this idea and demonstrate, in addition, that the binding of dPGS to proteins may be directly compared and modeled as the binding of DNA to various proteins.

### 3.2 Sulfated Polyglycerol as an Anti‐inflammatory Drug

**Figure 7:**
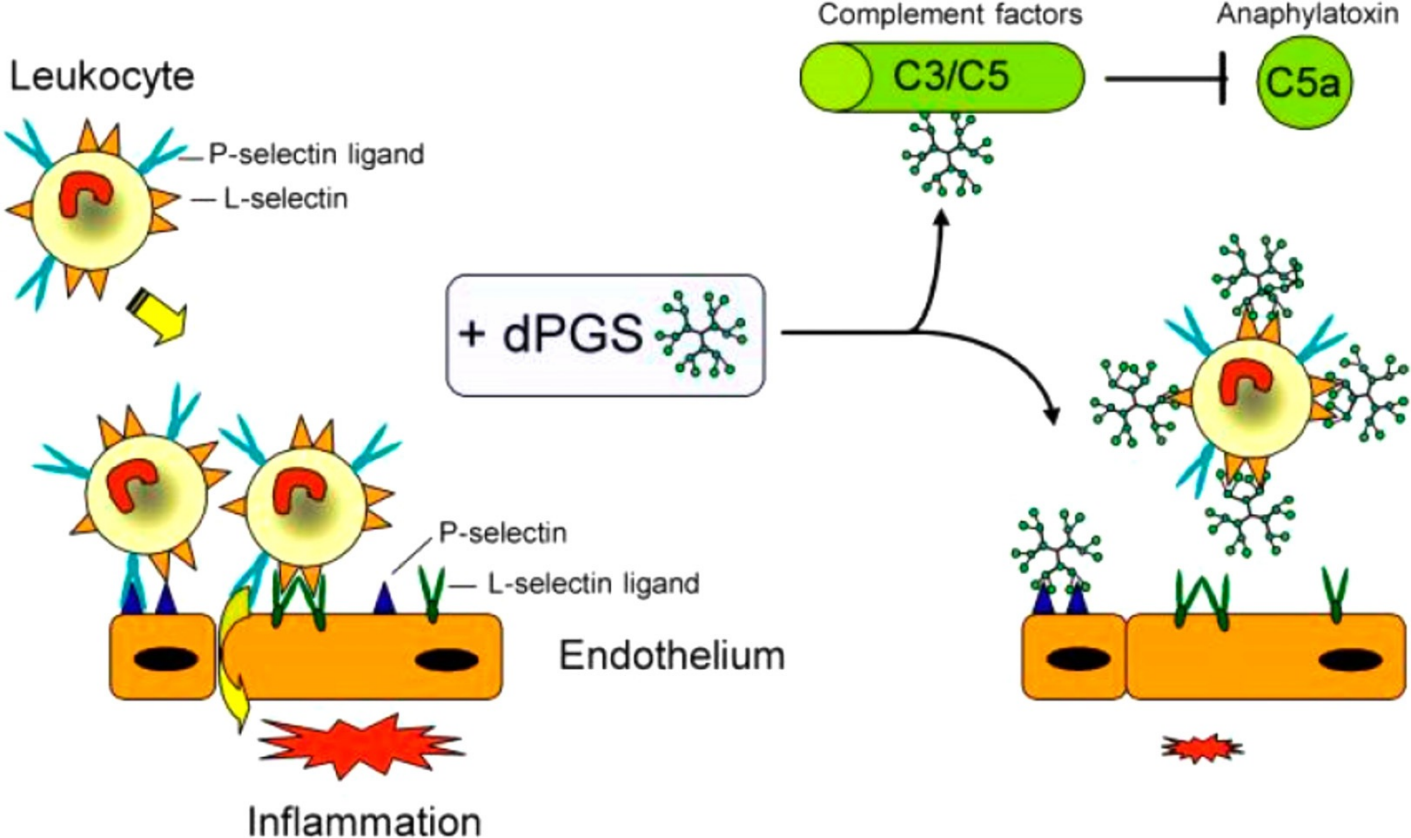
The anti‐inflammatory effect of dPGS. As shown by Dernedde et al.,^[36]^ dPGS inhibits an overwhelming inflammatory response and reduces the extravasation of leukocytes. dPGS targets the adhesion molecules L‐ and P‐selectin, while no binding to E‐selectin is observed. The same finding was made in our recent study by MD simulations.^[45]^ Thus, dPGS acts by preventing leukocyte extravasation through the binding of the selectins. Moreover, binding to complement factors C3 and C5 inhibits the formation of the proinflammatory anaphylatoxins. Here, the reduction of the C5a level decreases further leukocyte activation and recruitment. As a result, the adhesion cascade is balanced and contributes to initiate the healing process.^[196]^

As already mentioned in the Introduction, dPGS has a strong anti‐inflammatory effect.[^34^, ^36^, ^196^] Figure 7 shows the mode of action of dPGS: The recruitment of leukocytes to the sites of inflammation is an important step in the pathogenesis of acute and inflammatory diseases, which include hypersensitivity reactions and autoimmune diseases. This process is orchestrated by gradients of cytokines and chemokines and by distinct expression and activation of several family members of adhesion molecules, including selectins and integrins. Dernedde et al. demonstrated that dPGS binds to the positively charged amino acid residues (arginines) close to the carbohydrate binding pocket of L‐and P‐selectin with high affinity in the nanomolar range; no binding takes place with E‐selectin due to the absence of basic residues.^[36]^ As discussed above, these findings were recently directly proven by MD simulation of the interaction of these selectins: Counterion release was found to be the main driving force for binding, and the experimental binding constant of a third‐generation dPGS with L‐selectin^[78]^ could be reproduced by the simulation in a nearby quantitative fashion.^[45]^

It is important to note that ion‐specific effects may play an important role as well. Thus, Weinhart et al. analyzed the interaction of several polyglycerol‐based anions with L‐selectin.^[197]^ The strength of interaction increased in the order carboxylate (no inhibition)< phosphate< phosphonate≈sulfonate< bisphosphonate< sulfate. Hence, the electrostatic effect alone cannot be solely responsible for the strength of binding. Furthermore, Paulus et al. studied the effect of dPGS‐branching on the inhibition of inflammatory processes.^[191]^ It was found that a dPGS with a degree of branching of 60 % had a higher binding strength than a sulfated, perfect dendrimer characterized by a degree of branching of 100 %. More recently, biodegradable dPGS was shown to exhibit promising features for anti‐inflammatory applications, thus replacing heparin.^[196]^ All the results obtained so far clearly reveal charge–charge interactions to be the major driving force for binding.

In the meantime, several inflammation and tumor‐relevant proteins were identified as nanomolar binders for dPGS, such as interleukin‐1 (IL‐1), L‐selectin, P‐selectin, interleukin‐6 (IL‐6),^[198]^ lectin‐type oxidized low‐density lipoprotein receptor 1 (LOX‐1),^[199]^ and the complement factors C1q and C5a.^[200]^ The binding of dPGS is rather unspecific and does not necessarily depend on a unique protein structure. This is in contrast to species‐specific inhibitors that target ligand–receptor interactions with peptides, proteins, or antibodies. Thus, targeting by dPGS is much less sensitive due to evolution‐driven variations.

### 3.3 Complement Pathway

**Figure 8:**
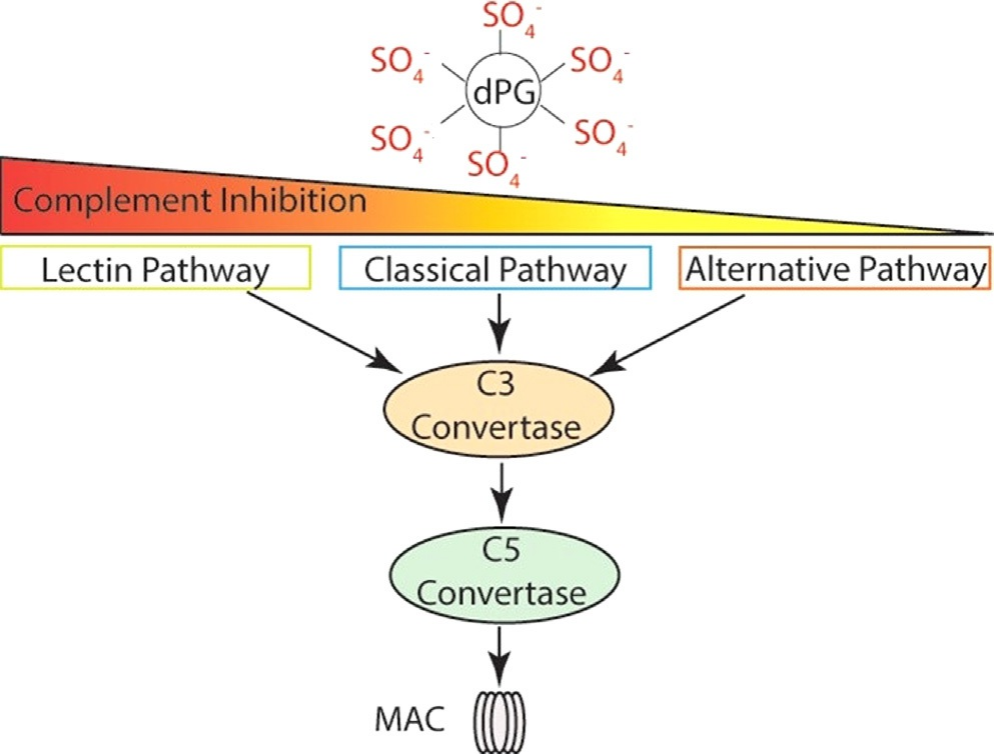
Complement pathway: dPGS interferes with the three pathways of complement activation and reduces formation of the membrane attack complex (MAC), which is a pore that is inserted into the cytoplasmic membrane and thereby leads to cell death. Reduced activity of the C3 and C5 convertase results in IC_50_ values of 60 nm (lectin pathway), 300 nm (classical pathway), and 900 nm (alternative pathway).^[200]^

The immune system of vertebrates consists of a combination of complex mechanisms that must be tightly regulated to act prompt and properly.^[201]^ Here, the innate immune system is the unspecific first line of defense against invading microorganisms and must act broadly to detect and eliminate pathogens. The complement system consists of a number of soluble blood proteins that are activated through a proteolytic cascade mechanism. Inhibition at distinct checkpoints paralyze the complement activation and are of importance in several pathologies characterized by dysregulated excessive activation, with sepsis being the most prominent disease.[^202^, ^203^] Recently, Silberreis et al. identified that dPGS targets the three different pathways of the complement cascade and that charge–charge interaction plays an important role to balance the activation (Figure 8).^[200]^ It was shown that dPGS binding to the complement factors C3 and C5 inhibits further processing and subsequent release of the anaphylatoxins C3a and C5a. In addition, charge‐dependent sequestration limits the anaphylatoxin function. Highly charged polyelectrolytes such as heparin and heparan sulfate were shown to act similarly,^[204]^ but by far not as effective as the synthetic polymer dPGS that binds the anaphylatoxin C3a with low micromolar affinity and C5a with nanomolar affinity.^[200]^ Thus, dPGS may be a promising candidate for a drug that counteracts an overshooting complement activation in sepsis and other diseases such as rheumatoid arthritis.[^205^, ^206^, ^207^]

### 3.4 Interaction of dPGS with Cellular Systems

**Figure 9:**
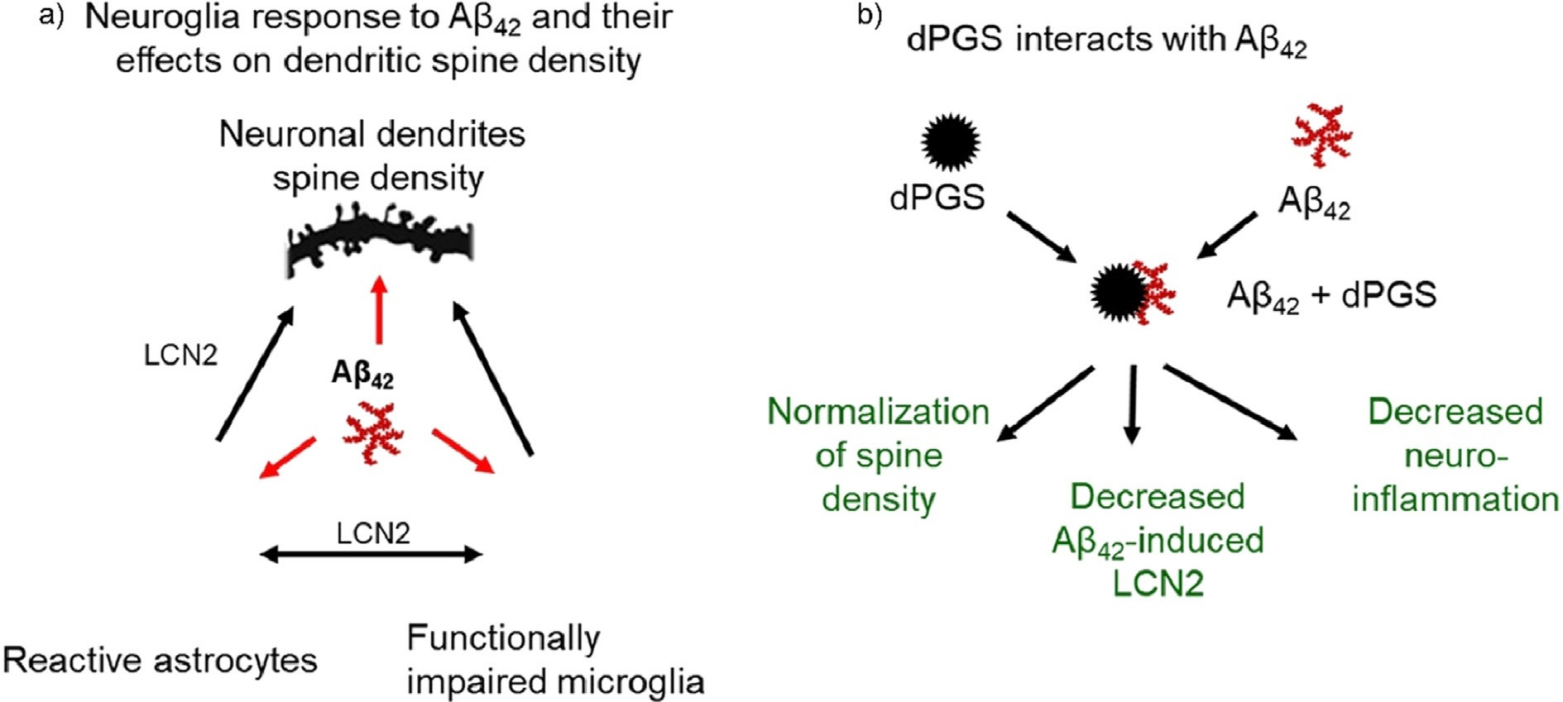
Modulatory effects of dPGS in neuroinflammation caused by Aß oligomers. a) Exposure of microglia to Aβ oligomers causes the activation of microglia and loss of dendritic spines in the hippocampal excitatory neurons. Hyperactive microglia activate astrocytes and these glial cells (reactive astrocytes) produce excessive amounts of lipocalin 2 (LCN2). LCN2 in combination with cytokines released from hyperactive microglia contribute to the impairment of synaptic functions. b) dPGS attenuates microglia hyperactivity, binds to Aβ_42_ and normalizes the number and function of dendritic spines.^[40]^

The effect of dPGS on neural cells was also investigated in models of endotoxemia caused by lipopolysaccharide (LPS) in primary neural cultures and in animals.^[198]^ Figure 9 shows the main findings in a schematic fashion: dPGS can reduce the negative impact of cytokines on neural brain cells through attenuation of the hyperactivity of microglia and lipocalin‐2 release from astrocytes. Enhanced microglia activation caused astrocyte activation, and dPGS was a powerful modulator of the cross‐talk between the microglia and astrocytes. dPGS directly bound to IL6, thereby preventing the binding of cytokine to its receptors and reduced the propagation of neuroinflammation. dPGS was internalized both by microglia and astrocytes in a concentration‐ and time‐dependent manner.

Aside from strong neuroglia activation by LPS, Aβ_42_ oligomers can also activate neuroglia but to a lesser extent.^[40]^ The mechanism of the dPGS action involved a direct binding of the Aß_42_ oligomers to dPGS, thus interfering with the formation of Aβ fibrils.^[40]^ The treatment with dPGS prevents the deleterious effects of oligomeric Aβ on dendritic spines at the excitatory synapses in the hippocampus and normalizes the neuroglia activity in this brain structure (Figure 9). Taken together, these studies suggest that dPGS is a valid candidate for therapeutic interventions in neurodegenerative disorders implicating neuroinflammation of the central nervous system.

Summing up the previous work related to dPGS and its application to various systems and medical problems, it becomes evident that the marked localization of the counterions on the surface of these dendritic structures provides the key for the understanding of the results: MD simulations together with experiments^[51]^ demonstrate that the high charge density on the surface leads to a surface concentration of counterions of the order of 1 m [see the discussion of Equation (3)]. Electrostatic interaction with proteins and more complicated systems will release a part of these surface‐bound ions into the bulk solution with a reduced ion concentration. In cells, this concentration is 150 mm, whereas the extracellular matrix is characterized by even lower salt concentrations. Binding will be brought about by entropic forces that work even under physiological salt concentrations. Evidently, this counterion release force is only one part of the free energy, other factors, such as the release of water molecules and hydrogen bonding, will come into play as well.

### 3.5 Polyelectrolyte Brushes

**Figure 10:**
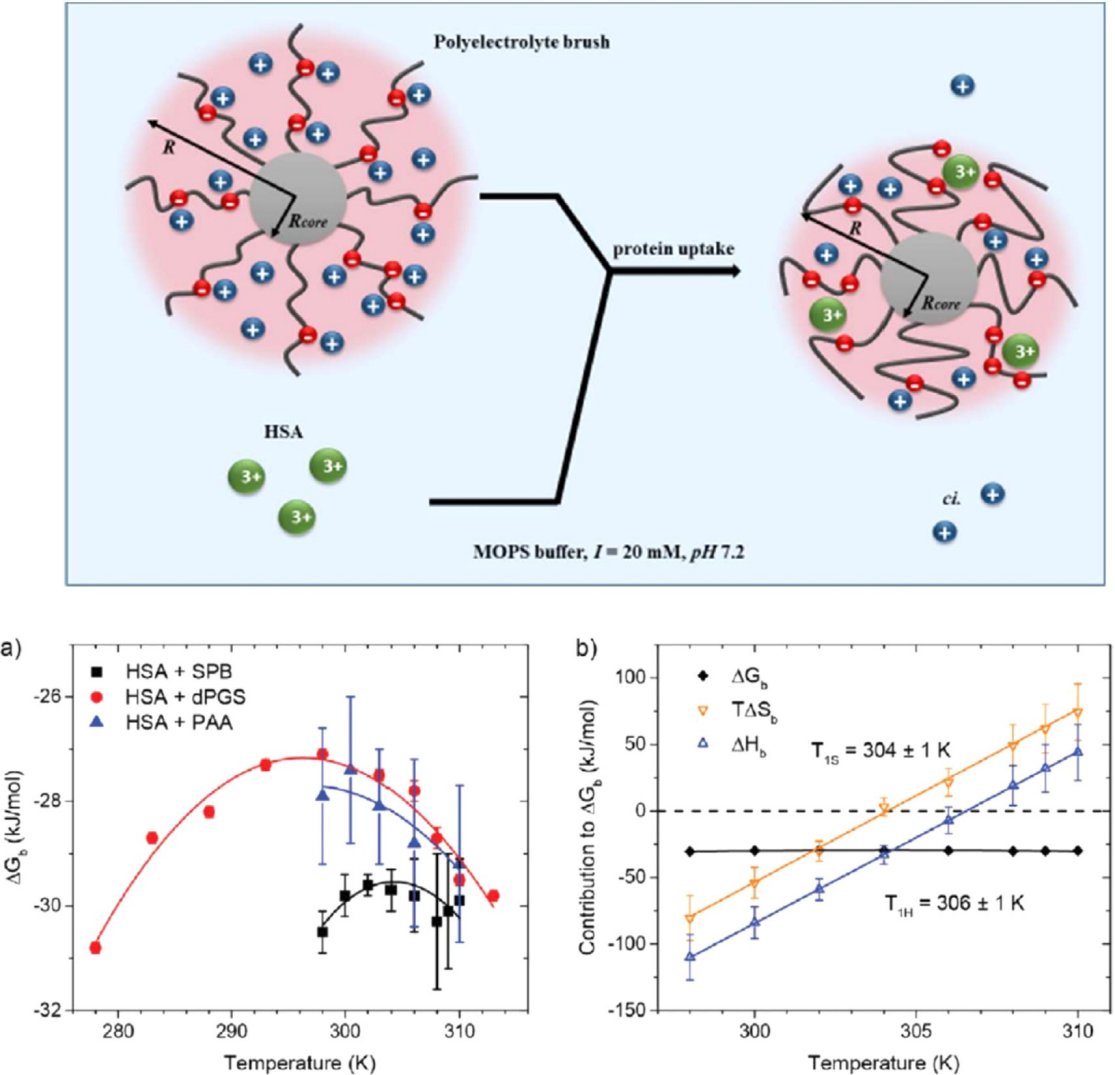
Uptake of proteins by a spherical polyelectrolyte brush (SPB).^[208]^ Top: The polyelectrolyte brushes consist of a solid polystyrene core (gray sphere) with a radius *R*_*h,core*_ between 50 and 100 nm. Onto its surface are grafted long chains of polyelectrolytes, for example, poly(acrylic acid). Red spheres on the PAA chains represent the negative charge of the acidic residues, while blue spheres represent the positive counterions. Nearly all of the counterions of the brushes are confined within the brush layer (osmotic brush). The protein molecules are represented by green spheres. Their uptake will lead to the release of a concomitant number of counterions. Adsorption of proteins by polyelectrolyte brushes is hence mainly entropy‐driven.[^109^, ^208^] Bottom: a) The Gibbs free energy of binding Δ*G_b_* of HSA to a spherical polyelectrolyte brush carrying long chains of poly(acrylic acid) (black squares) compared to the results for HSA binding to dPGS and of HSA interacting with linear chains of poly(acrylic acid). In all cases, the ITC‐determined Δ*G_b_* exhibits only a weak dependence on temperature, which is followed by a strong enthalpy–entropy compensation (EEC) shown in (b) for HSA interacting with a SPB: Both Δ*H_b_* as well as *T*Δ*S_b_* vary strongly with temperature, whereas Δ*G_c_* stays nearly constant because of the EEC.^[208]^

If long linear polyelectrolyte chains are appended to planar or curved surfaces, a polyelectrolyte brush results.[^110^, ^209^, ^210^, ^211^, ^212^] The brush limited is reached when the average distance between the grafted chains on the surface is smaller than their dimensions in solution.^[209]^ The interaction of these polyelectrolyte brushes with proteins has been the subject of a large number of studies, which have been reviewed recently.^[109]^ Hence, a brief discussion of this problem will suffice here. Figure 10 displays schematically the adsorption of proteins on spherical polyelectrolyte brushes. For a low ionic strength in solution, 95–98 % of the counterions are confined within the brush layer.[^110^, ^213^, ^214^] This confinement will lead to a high osmotic pressure within the brush layer and a concomitantly strong stretching of the polyelectrolyte chains.^[214]^ The uptake of proteins will lead to a partial release of these counterions, which is the main driving force for adsorption.[^111^, ^215^] At high ionic strength, on the other hand, the limit of a salted brush is attained.[^211^, ^213^, ^214^] at this limit, proteins can hardly adsorb on the brush layer. Moreover, adsorbed proteins will be released when going from a low ionic to a high ionic strength.[^216^, ^217^] The interaction of proteins with such a dense polyelectrolyte layer can, hence, be understood in terms of the counterion release force discussed above in Section 2.1. FTIR spectroscopic studies revealed that there is hardly any change in the secondary structure of the adsorbed proteins.[^218^, ^219^] The same conclusion could be drawn from the activity of adsorbed enzymes[^220^, ^221^] and from spectroscopic studies of the green fluorescent protein adsorbed on a spherical polyelectrolyte brush.^[217]^

It is interesting to note that the adsorption of proteins on spherical polyelectrolytes is accompanied by a marked enthalpy–entropy compensation, exactly in the way discussed for the dPGS/lysozyme system (see the discussion of Figures 5 and of 10 b,c). A recent study^[208]^ of the adsorption of human serum albumin on a spherical polyelectrolyte brush by ITC has revealed that the free energy of binding depends very little on the temperature, while the enthalpy and the entropy of adsorption vary linearly with temperature (Figure 10 c).^[208]^ Figure 10 b,c suggests that the strong enthalpy–entropy compensation is a general feature that always occurs when polyelectrolytes interact with proteins—from the complexes of DNA with proteins[^11^, ^49^, ^91^] to the binding of proteins to synthetic polyelectrolytes.[^54^, ^109^, ^208^] A fully quantitative theory of this effect, however, is still lacking.

### 3.6 Charged Networks

**Figure 11:**
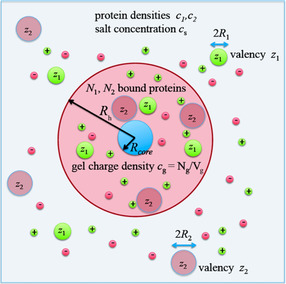
Modeling the competitive adsorption of proteins onto charged networks as exemplified by charged core–shell microgels.[^73^, ^74^] The shell consists of a charged network built up of hydrophilic chains. The network contains negatively charged monomer units, which lead to a charge density *c_g_*. The concentration of the counterions and the co‐ions within the network are regulated by the Donnan potential. The proteins are modeled by charged spheres with charge numbers *z*_1_ and *z*_2_, respectively, whereas the overall radii are given by *R*_1_ and *R*_2_, respectively. The uptake of proteins is governed by the interaction of the charged proteins with the Donnan potential of the network.^[73]^ The model can consider the competitive adsorption of several proteins onto the network.^[74]^ Here, two different proteins with effective charges *z*_1_ and *z*_2_ undergo competitive adsorption to the charged core–shell particle. The model leads to a fully quantitative understanding of the experimental results with four different proteins.^[74]^

Networks bearing charges have been a classical subject of polymer science and the first quantitative theory dates back to the classical paper of Michaeli and Katchalski from 1955.^[222]^ More recently, charged networks have been the subject of a series of comprehensive theoretical studies by Košovan, Holm, and co‐workers.[^76^, ^223^, ^224^, ^225^] It is fair to state that we now have acquired a very good physical modeling of these systems that helps us to understand their interaction with proteins. Figure 11 shows the main feature of charged networks exemplified for charged core–shell particles:[^73^, ^74^] The counterions are fully confined within the network and the total number of co‐ and counterions within the network is determined through the Donnan equilibrium. The Donnan potential determines the leading term for the interaction of charged entities such as proteins with the network. The decisive parameter for protein uptake is the difference in the ionic strength inside and outside the network and the overall charge of the proteins.^[74]^

There is a large number of experimental studies related to the uptake of proteins by charged networks, which started with a series of investigations by Kabanov, Zezin et al.[^55^, ^226^] Later studies include the work of Cohen‐Stuart and co‐workers.^[227]^ A more detailed discussion of these investigations is beyond the scope of the present Review. Here we only mention the studies by Yigit et al.,[^73^, ^74^] who investigated the uptake of various proteins by charged core–shell microgels and compared the findings to their theoretical model. In particular, Oberle et al.^[74]^ were able to show that this model can even predict the results of the competitive adsorption of two different proteins, thus demonstrating the power of a purely analytical model. Moreover, the difference in the free energy between the free and the adsorbed state of a protein can be used in Dynamic Density Functional Theory (DDFT) to model the kinetics of protein uptake into a network.^[75]^ DDFT is also capable of describing non‐monotonous effects in competitive adsorption^[228]^ (“Vroman effect”; cf. the discussion in Ref. [212]).

In a series of papers, Werner and co‐workers develop biocompatible highly charged hydrogels that can be used to adsorb and hence act as medical aids.[^27^, ^31^, ^56^, ^71^, ^229^] Glycosaminoglycan (GAG) based hydrogels with a varied GAG content and GAG sulfation pattern were prepared and applied to sequester cytokines. Cytokines are small proteins with various isoelectric points. Hydrogels containing GAGs with different sulfation patterns have been shown to adsorb cytokines, chemokines, and growth factors.[^31^, ^56^] Thus, networks containing defined GAG sequences can be employed, for example, for healing of chronically inflamed wounds by sequestering various cytokines. A review of this application and others has recently been provided by Werner and co‐workers.^[31]^

### 3.7 Virus Inhibition by Nanogels

As mentioned in Section 2.5, heparan sulfate (HS) moieties are located in the extracellular matrix and the glycocalyx. They are involved in the infection of many viruses through interaction with secondary receptors.[^37^, ^69^, ^79^, ^80^, ^145^] In general, viruses attach to and ultimately enter cells using multivalent interactions of viral ligands with receptors localized on the cell surface. Hence, nanoparticles of suitable size and that are highly charged can be used as multivalent receptors that compete with HS and thus block the docking of viruses on the cell surface.^[37]^ Sulfated nanogels with a size of 100–200 nm to match the virus size were synthesized and tested as antiviral agents. Flexibility of the cores turned out to be important because it resulted in a more effective shielding of the surface of the virus. Dey et al.^[37]^ demonstrated that, for example, HSV‐1 viruses are blocked by charge–charge interactions: The positively charged glycoproteins on the virus surface normally adhere to the negatively charged HS. Highly charged dPGS microgels can suppress this interaction by adhering to the virus and thus prevent the uptake of the virus by the cell. Thus, charge–charge interactions, most probably by counterion release, seems to be central for a clearer understanding of virus uptake and inhibition.

## 4 Complex Polyelectrolyte Architectures

In the last section of this Review, we now turn to systems with higher complexity. Here we deal with rather large polymeric structures that have been generated through the formation of covalent bonds or by self‐assembly, for example, micelles. These systems have been designed for special purposes, such as drug delivery, and must match a number of requirements: Low toxicity should be combined with high efficiency for targeting, for example, tumor cells. The polymeric scaffold with a size of 10–100 nm should be degradable for full clearance afterwards. The synthesis and analysis of complex architectures fulfilling these conditions certainly presents a great challenge, and the number of systems near to clinical use is still small. Here we choose two major problems in which polymeric systems have been applied successfully so far, namely drug delivery and anticoagulant reversal.

### 4.1 Drug Delivery

Micelles based on block copolymers with a charged block play a central role in this field. If the charged segments are characterized by a charge parameter *ξ*>1, counterion release will again be a major driving force for self‐assembly.[^88^, ^230^, ^231^] Polymersomes present another example for complex polymeric carrier systems.^[232]^ Much of this work has been reviewed recently by Kataoka and co‐workers,^[25]^ and so the present discussion of carrier systems will be focused more on recent studies using dPGS micelles.

Ideal polymeric drug carriers should, of course, fulfil two requirements: The micelles should be nontoxic and not interact with blood proteins. A strong adsorption of various blood proteins may lead to prompt immune reactions and opsonization (cf. the discussion in Refs. [233, 234, 235]). This problem has been addressed in many systems by a dense coating of poly(ethylene glycol) chains. Moreover, the micelles should carry their payload, for example, an anticancer drug, directly to the cancerous tissue in a highly specific manner. This requires concepts for targeting micelles and presents an important problem for present research (cf. the discussion of this point by Cabral et al.^[25]^).

### 4.2 Micelles for Tumor Targeting

**Figure 12:**
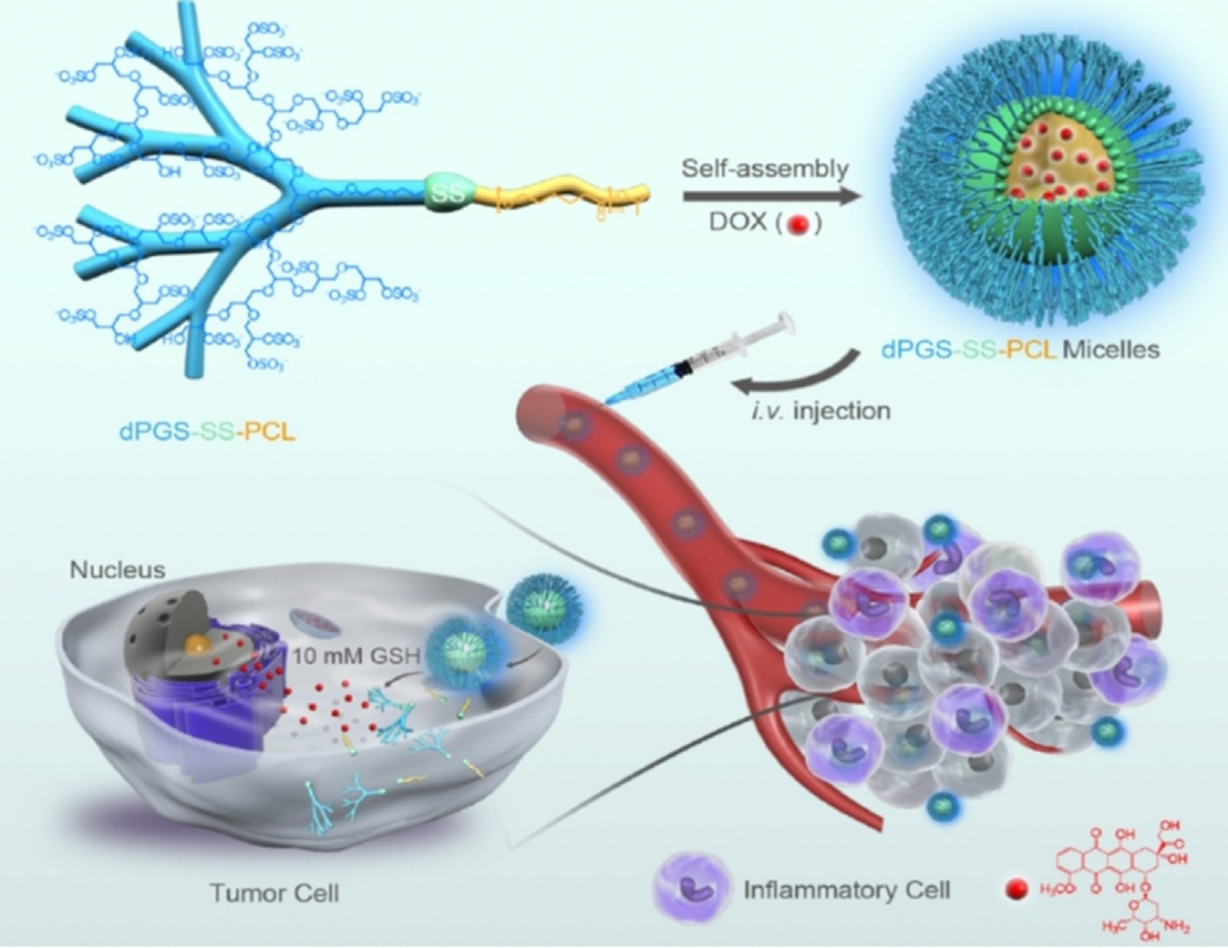
Micelles formed by the block copolymer from dPGS and poly(*ϵ*‐caprolactone) for active targeting of inflammation‐related tumor tissues.^[38]^ The micelles are assembled from a block copolymer consisting of a highly charged dPGS block and a hydrophobic poly(caprolactam) block. Both parts are interlinked by a sulfur bridge, which will be cleaved in the reductive environment of the cell. The drug doxorubicin (DOX) can be encapsulated in the hydrophobic core and, thus, brought to the infected tissue by intravenous injection. After uptake in the cells, the S‐S bridges are cleaved and the drug is released.

Dendritic dPGS‐based polymer micelle and the dPGS dendritic copolymer are highly potent candidates for the targeted delivery of poorly water‐soluble drugs. The extraordinary potential of such dPGS copolymer micelle formulations was first demonstrated by Zhong et al.,^[38]^ who used a disulfide‐bridged, cleavable dPGS‐SS‐PCL copolymer micelle (Figure 12) for the encapsulation of poorly water‐soluble dyes. This study provided the first demonstration of tumor‐targeted delivery and drug release for an intrinsic tumor‐affine polymer.^[38]^ To prove the applicability of dPGS copolymer micelle formulations of doxorubicin in vivo, Zhong et al. first investigated the elimination of doxorubicin from the blood in mice. Both cleavable and no‐cleavable micellar formulations delayed the elimination of doxorubicin from the blood (Figure 12 b). A factor of 10 increase in the bioavailability after a single parental application of doxorubicin was shown.

Further proof of concept was provided by the treatment of established human mammary MCF‐7 xenografts in nude mice. For this purpose, the hydrophobic anticancer drug doxorubicin was encapsulated within both the cleavable and noncleavable dPGS PCL copolymer micelle. The growth inhibition of human MCF‐7 mammary carcinoma cells in vitro was demonstrated with both formulations. We demonstrated that both cleavable and noncleavable dPGS copolymer micellar formulations of doxorubicin may increase the survival of tumor‐bearing mice compared to vehicle‐ or doxorubicin‐treated controls. However, stable long‐term survival in 100 % of implanted tumors was only achieved by repeated treatment with the doxorubicin‐loaded cleavable dPGS‐SS‐PCL micelles, which can release more antitumor drug specifically inside the cells of the tumor tissue.

### 4.3 Polycation‐Based Therapeutics for Polyanion Neutralization in Blood

Heparin‐based anticoagulant drugs (unfractionated heparin (UFH), low molecular weight heparins (LMWHs), and fondaparinux) are widely prescribed for prophylaxis and the treatment of thromboembolic disorders, as well as in surgeries.[^236^, ^237^] Despite its widespread use in clinics, a major limitation of this class of drug is a side effect of bleeding, which necessitates the need for antidotes which can neutralize their anticoagulant activity.[^42^, ^238^] To date, protamine is the only clinically approved antidote for UFH; however, it is not effective against all heparins.[^42^, ^238^] Protamine is a highly cationic polypeptide that interacts electrostatically with negatively charged heparins to form stable complexes, thereby providing antidote activity.^[157]^ Its cationic charge density and binding strength is not sufficient to generate a stable complex with LMWH or fondaparinux because of their low molecular weight and low degree of sulfonation (see Section 2).

**Figure 13:**
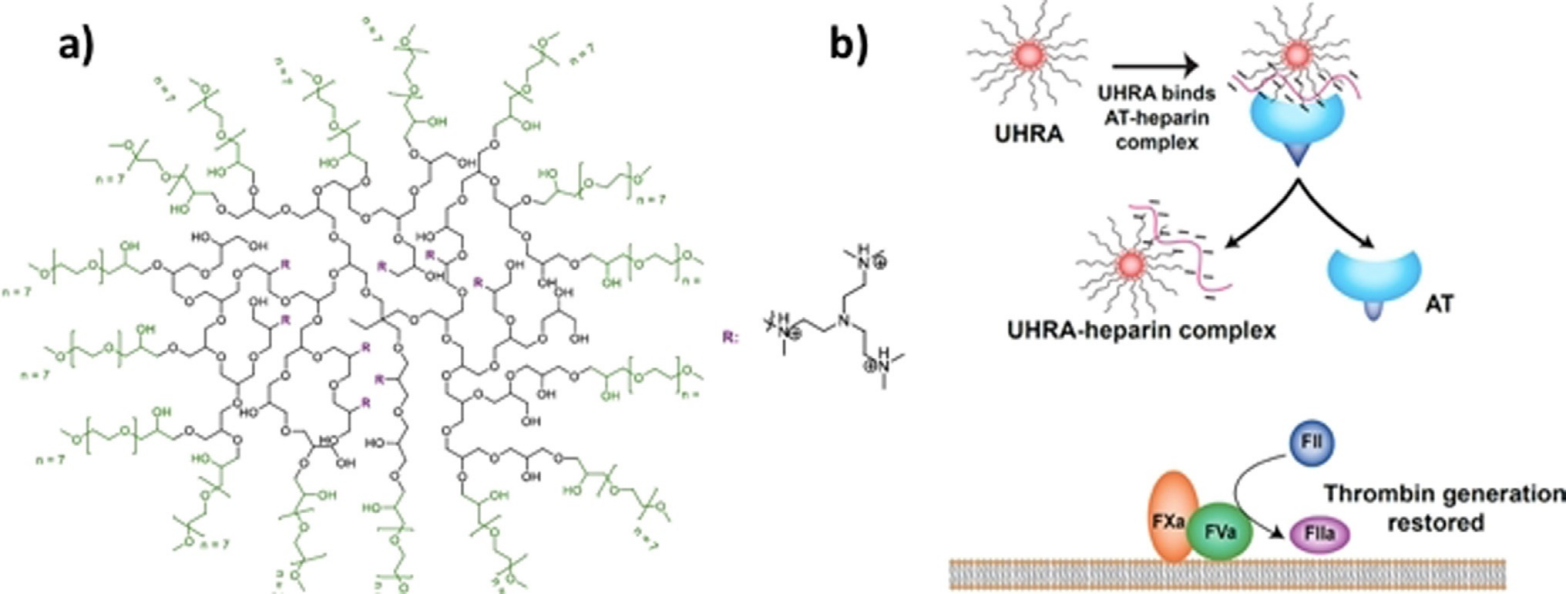
Design of UHRA and its interaction with heparin. a) Structure of UHRA and heparin binding groups (R). The chemical structure consists of an HPG‐based core and mPEG_350_ brush layer with hexamethylated tris(2‐aminoethylamine) as HBGs arranged in a multivalent fashion. b) The mechanism of the antidote action of UHRA. The antithrombin (AT) bound heparin complex responsible for the anticoagulant activity of polyanionic heparin is dissociated by interaction with the UHRA because of its high binding affinity. This process restores the generation of thrombin.[^41^, ^239^]

To overcome these deficiencies, the Kizhakkedathu group recently developed an UHRA, a synthetic nontoxic macromolecular heparin antidote capable of neutralizing all clinically available heparin‐based anticoagulants.[^41^, ^43^, ^239^, ^240^, ^241^, ^242^] Figure 13 a shows the chemical structure of the UHRA and its way of interacting with the antithrombin/heparin complex (Figure 13 b). The UHRA consists of a core of HPG and tertiary amine based heparin binding groups that acquire cationic charges at a physiological pH value. This core is protected by a shell of methoxypolyethylene glycol (mPEG) chains (brush layer). Unlike the naked cationic charges in protamine, the shielded dense cationic charge within the UHRA prevents its ionic interaction with endogenous anionic macromolecules in blood such as proteins (e.g. fibrinogen, coagulation factors) and cells (platelets, red blood cells). The mPEG brush layer offers sufficient entropic penalty to the incoming polyanions as a result of brush compression; thus, only those highly charged polyanions such as heparins can overcome such a barrier, thereby providing selectivity to UHRA.^[41]^ Thus, charge–charge interactions and probably counterion release play a major role in these processes.

## 5 Conclusion

The survey of investigations on natural and synthetic polyelectrolytes demonstrates that their interaction with proteins is largely dominated by charge–charge interactions. Section 2 shows that this interaction can be described by counterion release embodied in Equation (3). A similarly clear picture emerges from the studies done on dendritic polyelectrolytes, brushes, and networks summarized in Section 3. Specifically, systems based on dendritic polyglycerol sulfate (dPGS) are already applied in animal models for medical purposes, for example as anti‐inflammatory drugs, and the better understanding of the interaction of dPGS with proteins now achieved will certainly pave the way for many further applications. The situation is less clear for the more complex polyelectrolyte architectures discussed in Section 4. However, all the results obtained so far demonstrate the importance of charge–charge interactions, most probably related to counterion release. Moreover, all the investigations discussed here clearly reveal the importance of temperature as a decisive variable: In all the cases studied so far, a strong enthalpy–entropy compensation is observed. Further work is needed on this phenomenon and to explore its importance in living systems. The entire survey, however, clearly demonstrates that a much better understanding of charge–charge interactions is the key for the design of drugs based on polyelectrolytes.

At this point, we now suggest further work along the following lines: For a given architecture of a polyelectrolyte, two parameters are decisive: 1) ionic strength and 2) temperature. Hence, a meaningful study of the interaction of a polyelectrolyte system must always vary these two parameters. In particular, the investigation of potential drugs based on polyelectrolytes must always include experiments at 37 °C, which may lead to distinctly different results to the ones conducted at room temperature. Calorimetric studies carried out as a function of the salt concentration and temperature should be used to reveal and to design the strength and specificity of the interaction. Finally, the huge potential of MD simulations must be explored further. Here, a combination of simulations with studies on single molecules may be a new and very interesting avenue.[^243^, ^244^, ^245^, ^246^] Taken together, synthetic polyelectrolytes and systems derived therefrom are certainly highly promising candidates for the development of drugs.

## Conflict of interest

The authors declare no conflict of interest.

## Biographical Information

*Katharina Achazi studied biology at the FU Berlin, where she completed a Diploma (2006) at the institute for applied genetics and received her PhD at the Robert Koch Institute in the field of virology (2011). In 2012, after postdoctoral studies with Prof. M. Niedrig at the Robert Koch Institute, she joined the group of Dr. J. Dernedde at the Charité Berlin for research in collaboration with Prof. R. Haag at the FU Berlin. In 2013 she started a lab in the chemistry and biochemistry institute of the FU Berlin. Since 2016 she has also been head of the Optical Microscopy Unit of Core Facility BioSupraMol and since 2017 also the Microfluidics Unit*.

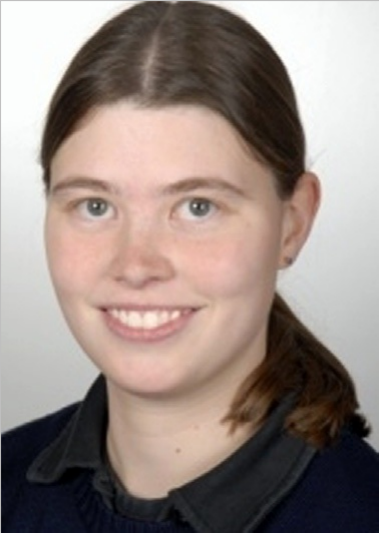

## Biographical Information

*Rainer Haag is Professor of Organic and Macromolecular Chemistry at FU Berlin. Since 2008 he has been head of SFB 765 “Multivalency as a Chemical Organization and Action Principle”. His research interests include dendritic polymers as highly functional polymer carriers for catalysis and macromolecular nanotransporters for DNA and drug delivery. In 2004, his group received the NanoFutur researcher award of the Federal Ministry of Science and Research. Together with the start‐up company Dendropharm, he received the Berlin‐Brandenburg Innovation Award 2016. Since 2019 he has been an elected member of the German Academy of Science and Engineering (acatech)*.

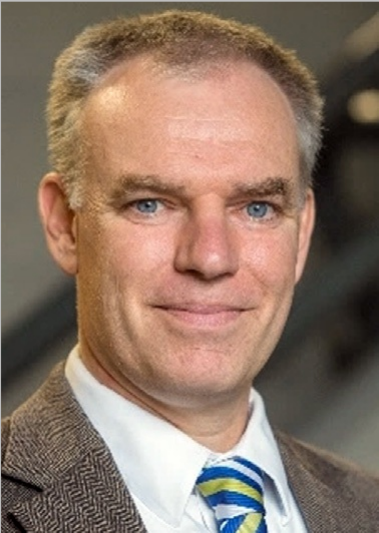

## Biographical Information

*Matthias Ballauff studied chemistry at the University of Mainz where he obtained his PhD in physical chemistry in 1981. After postdoctoral studies with P. J. Flory at Stanford University he joined the Max‐Planck‐Institut in Mainz as a research associate. 1990–2003 he was a professor at the University of Karlsruhe, and 2003–2009 he worked at the University of Bayreuth. In 2009, he joined the Helmholtz‐Zentrum Berlin and became a professor of experimental physics at the Humboldt University Berlin. After his retirement in 2019 he became a guest professor at the institute of chemistry and biochemistry of the Freie Universität Berlin*.

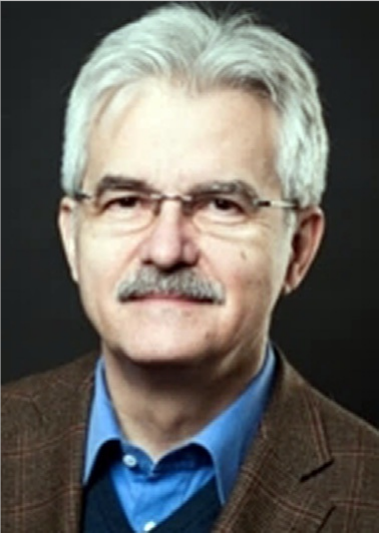

## Biographical Information

*Jens Dernedde studied biology with a focus on microbiology at FU Berlin, where he received his PhD in 1996. After postdoctoral studies in Berlin and Greifswald he joined in 1999 the Charité—Universitätsmedizin Berlin, which is the corporate member of of FU Berlin, Humboldt‐Universität zu Berlin, and the Berlin Institute of Health. Since 2012 he has had his own independent group within the Institute of Clinical Chemistry. His interests focus on inflammation and materials for distinct intervention in receptor–ligand recognition*.

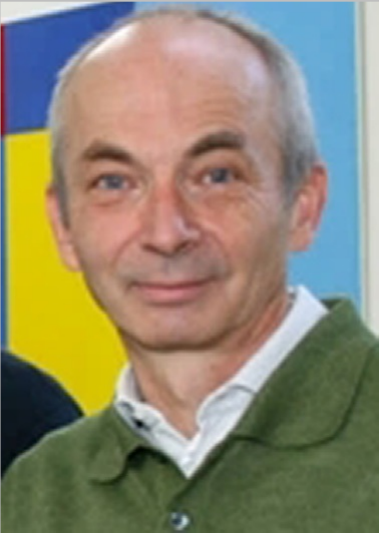

## Biographical Information

*Jayachandran Kizhakkedathu was born in Kerala, India. He completed PhD at the Indian Institute of Chemical Technology, Hyderabad in 2000 before moving to Canada. After postdoctoral studies with D. Brooks, he became an Assistant Professor in the Department of Pathology and Laboratory Medicine, University of British Columbia, Canada, in 2005. He is currently a Professor and Research Scholar of the Michael Smith Foundation for Health at the Centre for Blood Research. His research follows an interdisciplinary approach for the discovery of novel polymers as therapeutics, biomaterials, and technologies for biomedical and clinical use*.

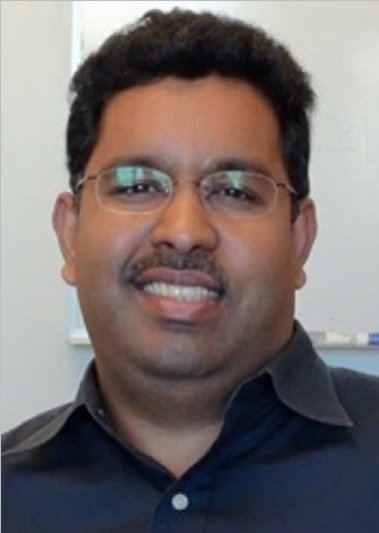

## Biographical Information

*Dusica Maysinger was born in Croatia. She earned her PhD at the University of Sothern California, Los Angeles, USA. After postdoctoral training in neuroscience at Max Planck Institute in Munchen, cell biology in Gottingen and Heidelberg, and Oxford, UK, she joined McGill University in Montreal, Canada, in 1984 where she is currently a full professor. Her area of expertise is chemical neuroscience and experimental neuropharmacology. Her research focuses on understanding nanostructured materials and their effects on different cell types*.

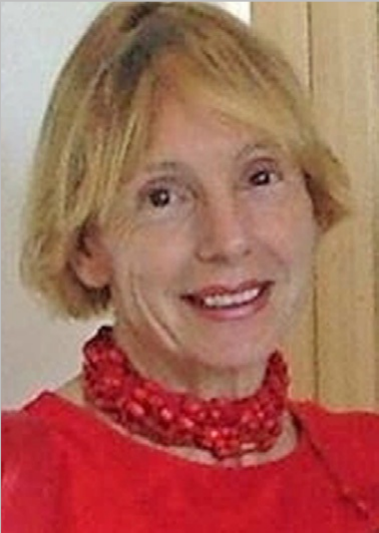

## Biographical Information

*Gerhard Multhaup studied Biology at the University of Cologne, Germany, where he graduated with a Diploma (1984) and received his PhD at the Institute for Genetics (1986). His first academic appointment was at the University of Heidelberg, Center for Molecular Biology (ZMBH). He was Full Professor at the FU Berlin for ten years (until 2012) before he was appointed as Chair of the Department of Pharmacology and Therapeutics at McGill University, Montreal, Canada. He holds a Canadian Research Chair Tier 1. His research covers structural and functional aspects of key proteins involved in Alzheimer disease and other disorders*.